# New 3*H*-1,2-Dithiole-3-thione derivatives: Design and synthesis, H_2_S-release profile, in vitro anticancer activity, and in silico multi-target assessment

**DOI:** 10.1007/s11030-026-11635-x

**Published:** 2026-07-26

**Authors:** Semra Altunsoy, Yakup Berkay Yılmaz, Tuğba Güngör, Hazal Nazlıcan Atalay, Mine Isaoğlu, Serdar Durdağı, Mehmet Ay, Tugba Boyunegmez Tumer

**Affiliations:** 1 Graduate Program of Chemistry, School of Graduate Studies, Çanakkale Onsekiz Mart University, Çanakkale, Turkey; 2 Graduate Program of Molecular Biology and Genetics, School of Graduate Studies, Çanakkale Onsekiz Mart University, Çanakkale, Turkey; 3 Natural Products and Drug Research Laboratory, Department of Chemistry, Faculty of Science, Çanakkale Onsekiz Mart University, Çanakkale, Turkey; 4 Computational Biology and Molecular Simulations Laboratory, Department of Biophysics, School of Medicine, Bahçeşehir University, İstanbul, Turkey; 5 Lab for Innovative Drugs (Lab4IND), Computational Drug Design Center (HİTMER), Bahçeşehir University, İstanbul, Turkey; 6 Department of Pharmaceutical Chemistry, School of Pharmacy, Bahçeşehir University, Beşiktaş, İstanbul, Türkiye; 7 Quantitative System Biology Lab, Faculty of Medicine, Biruni University, İstanbul, Turkey; 8 Department of Molecular Biology and Genetics, Faculty of Science, Çanakkale Onsekiz Mart University, Çanakkale, Turkey; 9 Department of Medical Biotechnology, Faculty of Biochemistry, Biophysics and Biotechnology, Jagiellonian University, Kraków, Poland

**Keywords:** 1,2-Dithiole-3-thione, Anticancer, Anti-inflammatory, Molecular docking, Molecular dynamics simulations, Binary QSAR modeling, SAR

## Abstract

Cancer continues to be a leading cause of global mortality, highlighting the ongoing need for novel anticancer compounds that offer high efficacy with improved side effect profiles. In the present study, a series of 3*H*-1,2-dithiole-3-thione derivatives (**DTT-S1-18**) were synthesized as promising anticancer agents, and the structures of products were confirmed by spectral techniques. H_2_S-releasing experiments showed that most of the compounds released higher amounts of H_2_S slowly over time compared to standard **ADT-OH**. All compounds were tested for antiproliferative activity on HT-29, PC-3, MCF-7, and HUVEC cell lines. Compounds **DTT-S6** (3-nitrophenyl derivative) and **DTT-S8** (methionine derivative) have the lowest IC_50_ values of 41.6 and 38.9 µM on the MCF-7 cell line, respectively. Based on the wound healing and colony formation assays performed in MCF-7 cells, the wound areas were not significantly changed after treatment with compounds **DTT-S6** and **DTT-S8**, whereas compound **DTT-S8** at double IC_50_ dose inhibited colony formation by 81.82%. In addition, molecular docking, MD simulations, MM/GBSA binding free energy calculations, and binary QSAR analyses were performed to explore the potential target interactions and predicted activity profiles of the synthesized compounds toward inflammation-related proteins, including COX-1, COX-2, 5-LOX, and iNOS, thereby supporting the development of mechanistic hypotheses for future validation. Furthermore, structure–activity relationship (SAR) analyses were conducted to correlate the structural characteristics of the synthesized compounds with their H_2_S releasing potential and biological profiles. Overall, this work integrates experimental anticancer evaluation with computational pathway and structure-based cancer/inflammation analyses to characterize novel DTT-based H_2_S donors. The findings identify particularly compound **DTT-S8**, as a promising in vitro anticancer candidate, while the computational results suggest a putative involvement of inflammation-related targets, particularly the COX-2/5-LOX axis, which requires direct biochemical and cellular validation.

**Supplementary Information:**

The online version contains supplementary material available at https://doi.org/10.1007/s11030-026-11635-x.

## Introduction

Hydrogen sulfide (H_2_S) is an endogenously produced gasotransmitter that regulates diverse physiological processes including redox homeostasis, inflammation, angiogenesis, and cell survival. In mammalian tissues, H_2_S biosynthesis is primarily mediated by cystathionine β-synthase (CBS), cystathionine γ-lyase (CSE), and 3-mercaptopyruvate sulfurtransferase (3-MST) [1–3]. Dysregulation of H_2_S signaling has been associated with multiple pathological conditions such as cardiovascular, metabolic, neurological diseases, and cancer [1, 4].

In cancer biology, H_2_S exhibits a condition-dependent role. Cancer cells frequently upregulate H_2_S-producing enzymes to enhance mitochondrial bioenergetics and ATP generation, thereby supporting tumor growth and progression [1, 3, 5]. Conversely, exogenous H_2_S donors can exert antiproliferative and pro-apoptotic effects depending on donor type, concentration, and cancer cell context, highlighting the fine balance between tumor-promoting and tumor-suppressive actions of H_2_S [3, 6].

**Fig. 1:**
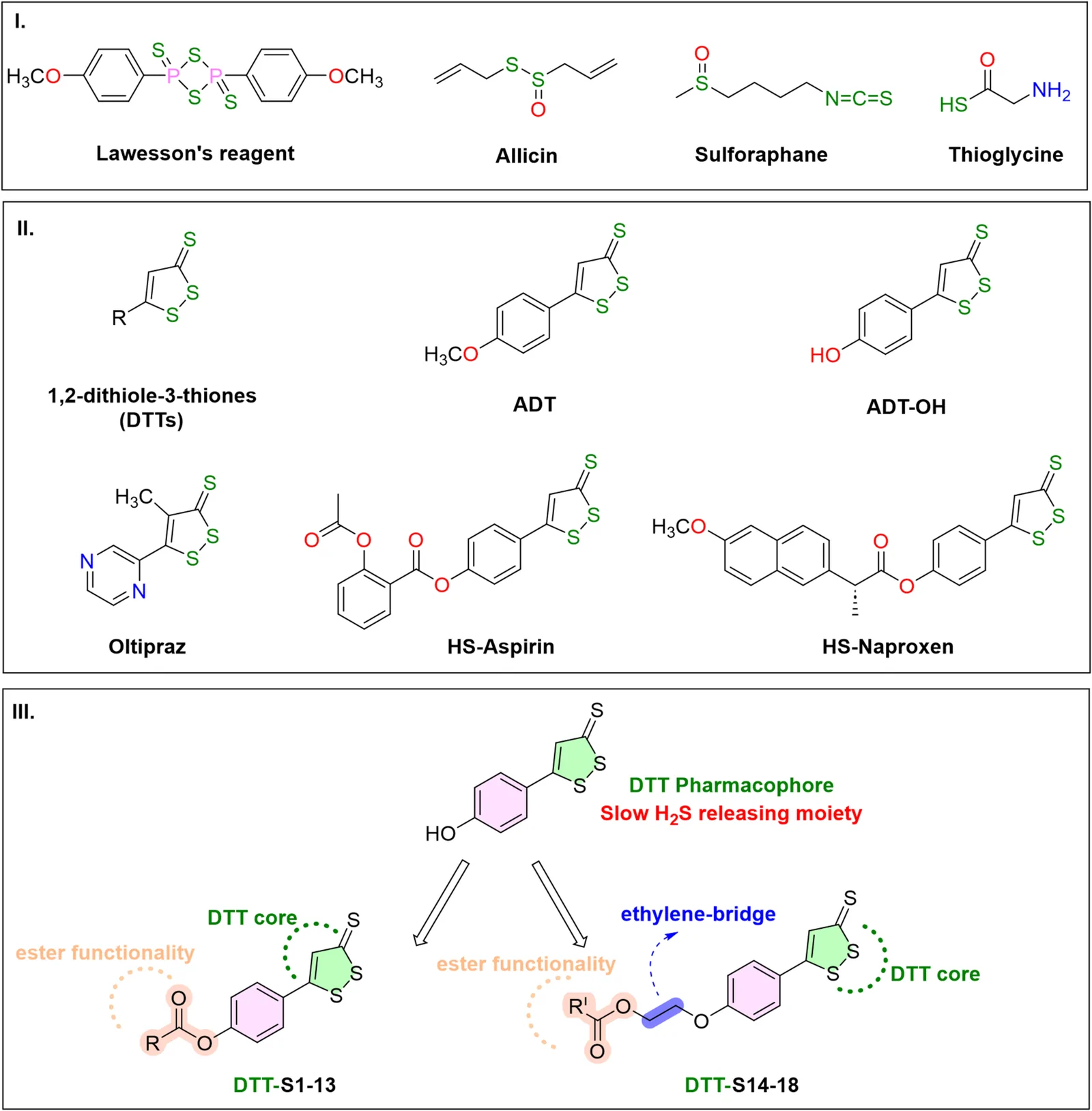
Overview of H_2_S donors. **I** Representative examples of synthetic and natural H_2_S donors. **II** DTT-based scaffolds and pharmacologically active hybrids. **III** Design strategy and general structures of the new target compounds **DTT-S1-18** in this study

Driven by these observations, extensive medicinal chemistry efforts have focused on developing synthetic H_2_S-releasing compounds, particularly for inflammation- and cancer-related indications. Broad reviews consistently emphasize anti-inflammatory and anticancer applications as the primary drivers of H_2_S donor design, with many scaffolds optimized to modulate oxidative stress, NF-κB signaling, COX/iNOS activity, and apoptosis [1–3]. Among these scaffolds, 3*H*-1,2-dithiole-3-thiones (DTTs) represent a well-established class of slow-releasing H_2_S donors with documented pharmacological relevance [7]. Apart from DTTs, inorganic sulfide salts such as Na_2_S and NaHS, Lawesson’s reagent and analogs such as GYY4137, sulfur-containing natural compounds such as allicin/garlic, sulforaphane/cruciferous vegetables, some amino acids such as thioglycine and synthetic organic compounds such as arylthioamides, acyl perthiols, isothiocyanates, and thiocarbamates are well-known H_2_S donors (Fig. 1). These donors release H_2_S through the mechanisms of hydrolysis, thiol activation, light activation, enzyme triggering, and pH control [8, 9].

On the anti-inflammatory side, the development of H_2_S-releasing non-steroidal anti-inflammatory drugs (NSAIDs) emerged from the need to reduce gastrointestinal toxicity associated with conventional NSAIDs. NSAID-induced suppression of CSE activity and increased leukocyte adherence are key contributors to gastrointestinal injury [10, 11]. H_2_S counteracts these effects by inhibiting leukocyte adhesion, suppressing pro-inflammatory cytokine production, and regulating redox-sensitive pathways such as NF-κB and mitogen-activated protein kinases (MAPKs) [12–14]. Accordingly, hybrid molecules combining NSAIDs with H_2_S-releasing moieties have demonstrated enhanced anti-inflammatory efficacy with improved gastrointestinal safety, exemplified by ATB-337 and ATB-346 [15–16]. Similarly, NOSH-aspirin, integrating both nitric oxide (NO) and H_2_S donors, retained the pharmacological effects of aspirin while reducing gastrointestinal toxicity and improving chemopreventive activity [17–18].

Beyond inflammation, DTT-based H_2_S donors have attracted growing interest in their anticancer properties (Fig. 1). ADT-OH, the main metabolite of ADT, releases H_2_S upon hydrolysis and serves as a key pharmacophore in numerous hybrid molecules. Oltipraz, a synthetic DTT derivative, is a well-known Nrf2 activator (nuclear factor erythroid 2-related factor 2) used as a chemoprotective agent against liver cancer and as an inhibitor of HIV-1 replication [19–21]. Numerous ADT-NSAID hybrids have demonstrated synergistic antiproliferative activity and reduced gastrointestinal toxicity through controlled H_2_S release [9, 22–24].

Experimental studies further support the anticancer potential of H_2_S donors. Exogenously delivered H_2_S from NaHS was shown to stimulate intracellular Ca^2+^ signaling in human metastatic colorectal cancer cells (mCRCs) [1]. Chattopadhyay and colleagues reported that H_2_S-releasing NSAID hybrids inhibited proliferation across multiple cancer cell lines including colon, breast, pancreatic, prostate, lung, and leukemia with markedly greater potency than the parent NSAIDs [25]. Moreover, co-administration of NaHS with paclitaxel enhanced antitumor efficacy while reducing oxidative stress in ovarian cancer models [26].

In this study, we primarily investigated the anticancer activity of eighteen new DTT-based H_2_S donors **(DTT-S1-18)** which were designed, synthesized via esterification reactions, and structurally characterized (Fig. 1). Their H_2_S-releasing capacities were evaluated, and antiproliferative effects were experimentally assessed using SRB-based cytotoxicity assays in HT-29, PC-3, and MCF-7 cancer cell lines, as well as in non-tumorigenic HUVEC endothelial cells. Wound-healing and colony-formation assays were further employed to characterize the effects of selected compounds on cancer cell migration and long-term survival. To analyze mechanistic hypotheses that may help explain the observed biological activities and possible interactions with inflammation- and cancer-related targets, extensive in silico analyses were conducted. These comprised molecular docking, molecular dynamics (MD) simulations, and Molecular Mechanics/Generalized Born Surface Area (MM/GBSA) calculations, meticulously mapping the binding profiles of the compounds within different targeting strategies for inducible nitric oxide synthase (iNOS), cyclooxygenase-1 (COX-1), cyclooxygenase-2 (COX-2), and both catalytic and allosteric sites of 5-lipoxygenase (5-LOX) enzymes. These computational studies were intended to support mechanistic interpretation rather than to experimentally validate anti-inflammatory activity. Furthermore, binary quantitative structure-activity relationship (QSAR) models, utilizing the MetaDrug™ platform, were employed for the prediction of potential activity and toxicity profiles. Our computational findings suggest that the most promising candidates may interact with the COX-2/5-LOX axis, although direct experimental confirmation of COX-2 and 5-LOX inhibition remains necessary. This integrated approach suggests a possible association between slow H_2_S release, predicted interactions with inflammation-related targets, and the observed anticancer activity. However, the underlying mechanism remains to be experimentally validated.

## Results and discussion

### Chemistry

**Scheme 1:**
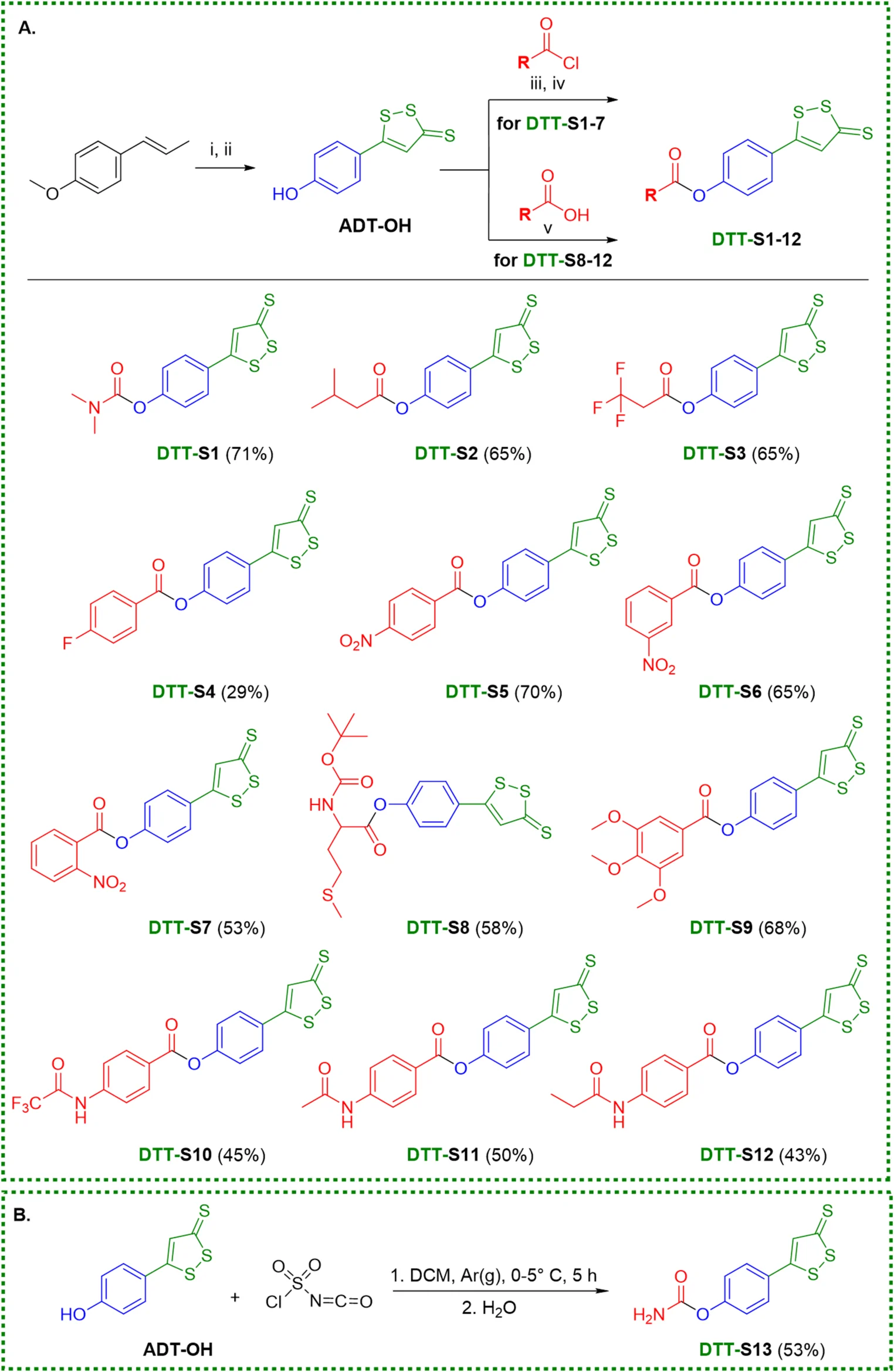
The synthetic route of compounds **DTT-S1-13** Note: Reagents and conditions: **(i)** S_8_, DMF, reflux, 8 h; **(ii)** Pyridine.HCl, MW, 200 W, 200 °C, 7 min; **(iii)** Pyridine, rt, 3 h (for comp. **DTT-S1**); **(iv)** Et_3_N, CHCl_3_, rt, 2–6 h (for comp. **DTT-S2-7**); **(v)** DCC, DMAP, DCM, rt, 2–8 h (for comp. **DTT-S8-12**).

Ester derivatives (**DTT-S1-13)** or ethylene-bridged ester derivatives (**DTT-S14-18)** of the 5-phenyl-3*H*-1,2-dithiole-3-thione skeleton were synthesized by esterification reactions using the corresponding carboxylic acids or acyl chlorides bearing different functional groups. Scheme 1 outlines the synthesis of starting material **ADT-OH** (5-(4-hydroxyphenyl)-3*H*-1,2-dithiole-3-thione), and final products **DTT-S1-13**. The starting material **ADT-OH** was synthesized over two steps according to the literature methods [27–28]. In the first step, the mixture of anethole and S_8_ was refluxed in DMF for 8 h, and **ADT-OMe** (5-(4-methoxyphenyl)-3*H*-1,2-dithiole-3-thione) was obtained with 60% yield [27]. In the second step, which involved the conversion of an aromatic ether to a phenol derivative, **ADT-OH** was obtained in a yield of 77% by reacting **ADT-OMe** with pyridine hydrochloride for 7 min under microwave irradiation [28]. The synthesis of target compounds **DTT-S1-12** was achieved with the esterification reaction of **ADT-OH** and various acyl chloride/carboxylic acid derivatives. While compound **DTT-S1** was obtained with dimethyl carbamoyl chloride in pyridine as base and solvent with 71% yield, compounds **DTT-S2-7** were synthesized from **ADT-OH** and different aliphatic/aromatic acyl chlorides (isovaleryl chloride, 3,3,3-trifluoropropanoyl chloride, 4-fluorobenzoyl chloride, 4-nitrobenzoyl chloride, 3-nitrobenzoyl chloride and 2-nitrobenzoyl chloride) with triethylamine in chloroform at room temperature in the yield of 29–70%. Also, the syntheses of ester derivatives **DTT-S8-12** were performed starting from **ADT-OH** and carboxylic acids (Boc-Met-OH, 3,4,5-trimethoxybenzoic acid, 4-(2,2,2-trifluoroacetamido)benzoic acid, 4-acetamidobenzoic acid and 4-propionamidobenzoic acid) with DCC and DMAP in dichloromethane for 2–8 h at room temperature in the yield of 43–68% (Scheme 1A). Carbamate derivative **DTT-S13** was obtained from **ADT-OH** and chlorosulfonyl isocyanate (CSI) in DCM in an ice bath for 5 h with a moderate yield (53%) (Scheme 1B).

**Scheme 2:**
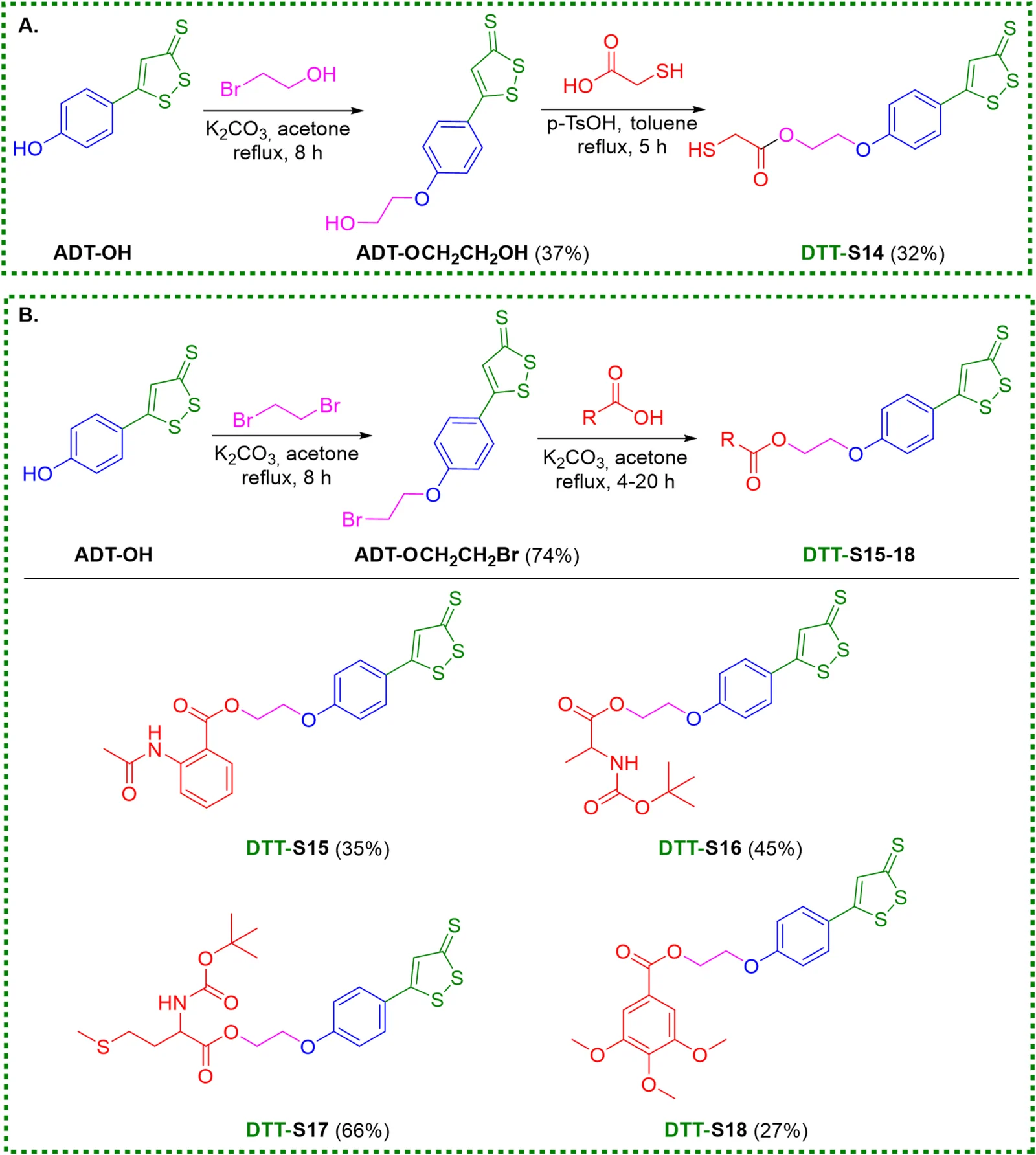
The synthetic route of compounds **DTT-S14-18**

The syntheses of compounds **DTT-S14-18** are summarized in Scheme 2. Compound **DTT-S14** was synthesized in two steps. Firstly, intermediate **ADT-OCH**_**2**_**CH**_**2**_**OH** was obtained according to the literature method [29]. In the second step, the mixture of **ADT-OCH**_**2**_**CH**_**2**_**OH**, thioglycolic acid, and catalyst *p*-TsOH was refluxed in toluene for 5 h with a Dean-Stark apparatus to give the final product **DTT-S14** (Scheme 2A). As seen in Scheme 2B, the substitution reaction of **ADT-OH** and 1,2-dibromoethane at basic conditions afforded the intermediate **ADT-OCH**_**2**_**CH**_**2**_**Br** [30] which was subsequently converted to the final ethylene-bridged ester products **(DTT-S15-18)** using carboxylic acids (2-acetamidobenzoic acid, Boc-Ala-OH, Boc-Met-OH and 3,4,5-trimethoxybenzoic acid) in the presence of potassium carbonate in acetone with 27–66% yields.

The structures of all products (**DTT-S1-18**) were verified by FT-IR, ^1^H NMR, ^13^C NMR, and MS spectroscopic analyses (see Experimental Part and Supplementary Information). In the IR spectra of compounds **DTT-S1-18**, the C = O stretching, which is the characteristic peak of the ester functional group, gives a strong peak in the range of 1697–1763 cm^− 1^. Also, the C = S double bond and C-S single bond in the sulfur-containing heterocycle were observed at 1156–1186 cm^− 1^ and 663–688 cm^− 1^, respectively. In the ^1^H NMR spectra of the products, two sets of protons with 2 H integration belonging to the *p*-disubstituted benzene appeared as doublet signals in the ranges of 7.84–8.04 ppm and 7.06–7.54 ppm, with a coupling constant of 8.2–8.9 Hz. The only proton in the sulfur heterocycle resonated as a singlet at 7.74–7.86 ppm chemical shift range. In the ^13^C NMR spectra of the compounds, the ester carbonyl carbon was observed in the range of 171.1-174.2 ppm, while the thiocarbonyl C = S carbon was observed in the range of 215.2-216.1 ppm. In addition to standard spectroscopic techniques, LC-MS analyses confirmed the molecular masses of final products.

### H_2_S releasing capacities of compounds DTT-S1-18

**Fig. 2:**
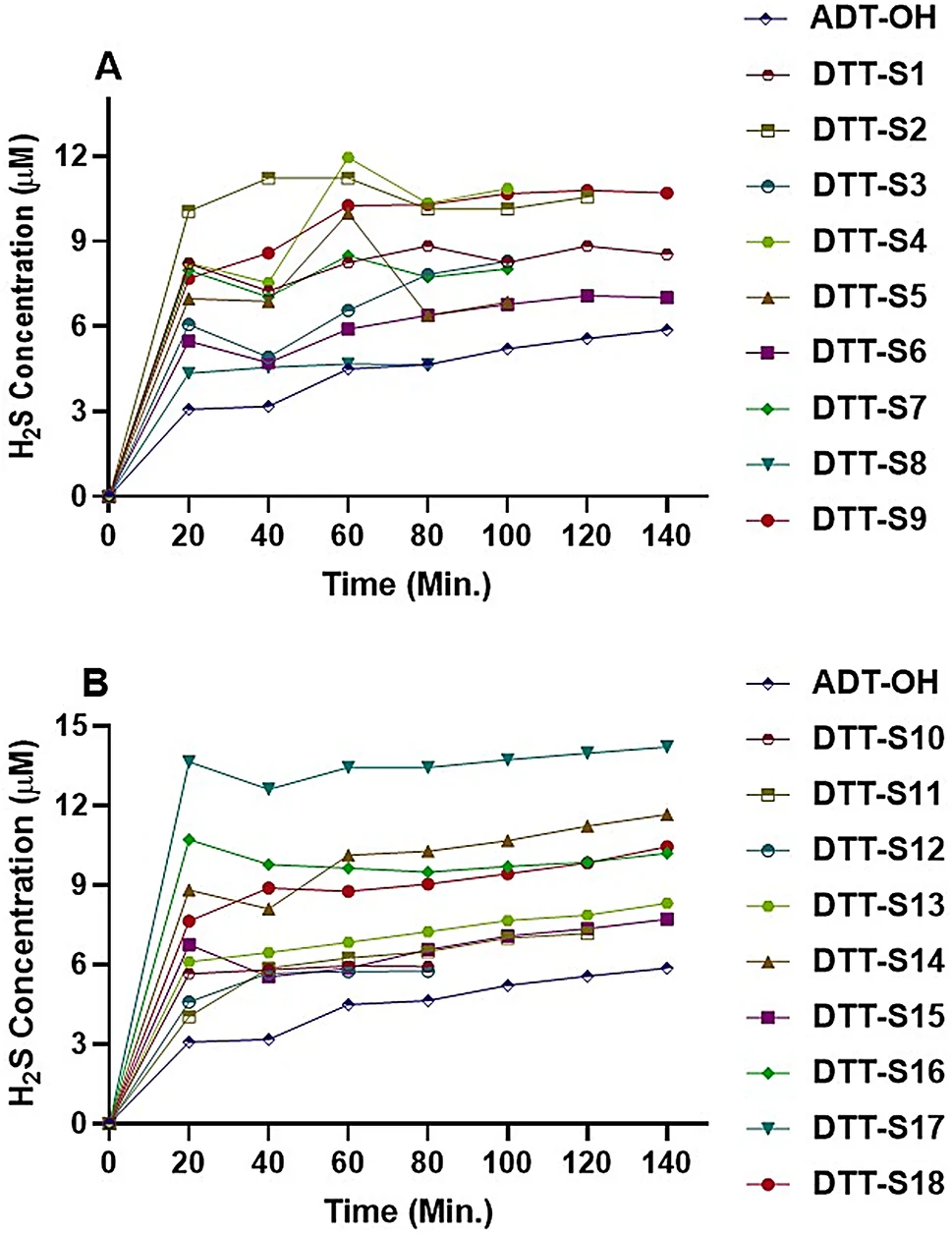
The amount of H_2_S released over time by (A) **ADT-OH** and compounds **DTT-S1-9 (B)** **ADT-OH** and compounds **DTT-S10-18**. Values represent the mean of three independent experiments (*n* = 3)

H_2_S is considered a physiological mediator and low steady-state levels of H_2_S are produced and maintained in the cells by several enzymes [31]. It has various distinct properties in the cellular processes such as mitochondrial function, homeostasis, inflammation, and inflammatory signaling [32–33]. Slow H_2_S release is considered favorable for compounds to mimic H_2_S release in the cells and prevent the loss of therapeutic activity with the sudden release, the latter may also have detrimental effects on the cells due to toxicity [34–35]. Although there are controversies about H_2_S release for its therapeutic activities on cancer cells, one explanation is the importance of H_2_S-donor compounds rather than H_2_S activity alone. Cancer proliferation is inhibited by slow H_2_S release rather than fast release, thereby losing biological activity immediately in the latter part [36–37]. Therefore, the H_2_S release capacities of compounds **DTT-S1-18** were assessed before antiproliferative activities. H_2_S release capacities of compounds **DTT-S1-18** were given in Fig. 2 along with standard **ADT-OH**, which was the starting material of final products. All compounds showed slow H_2_S release over time. **ADT-OH** released 6.75 µM of H_2_S after a 4-hour period. All compounds released higher amounts of H_2_S compared to **ADT-OH** except compound **DTT-S8**, which released 4.64 µM of H_2_S (Fig. 2A). Among all the compounds, compound **DTT-S17** showed the highest release with 13.38 µM of H_2_S (Fig. 2B).

### Anticancer activities of compounds DTT-S1-18

Selecting effective doses is essential for evaluating the anticancer activity of lead compounds. To this extent, IC_50_ values for the antiproliferative activity of all the H_2_S-releasing compounds were assessed on the HT-29, MCF-7, and PC-3 cancer cell lines with the SRB method alongside the HUVEC healthy endothelial cell line. As seen in Table 1, among all the compounds, **DTT-S6** and **DTT-S8** showed the most cell growth inhibition on the MCF-7 cell line with IC_50_ values of 41.61 and 38.87 µM, respectively (Supplementary Fig. S108). However, doxorubicin remained the most potent compound for cell growth activity on all the cell lines, including HUVEC (Table 1). An important consideration in the development of anticancer agents is their selectivity toward cancer cells relative to normal cells. Therefore, the antiproliferative activities of the synthesized compounds were also evaluated against the HUVEC healthy endothelial cell line. Assessment of cytotoxicity toward healthy HUVEC endothelial cells revealed limited selectivity of the tested compounds. Compound **DTT-S6** exhibited an IC_50_ value of 32.77 µM in HUVEC cells, which was lower than that observed in MCF-7 cells (41.61 µM), indicating a lack of cancer-cell selectivity. In contrast, compound **DTT-S8** showed a higher IC_50_ value in HUVEC cells (47.86 µM) than in MCF-7 cells (38.87 µM), corresponding to a selectivity index of 1.23. Although there is a modest preference toward MCF-7 cells, the degree of selectivity remains limited. Nevertheless, compound **DTT-S8** demonstrated moderate antiproliferative activity against MCF-7 cells and may serve as a preliminary lead compound for further structural optimization aimed at improving both anticancer potency and cancer-cell selectivity (Supplementary Fig. S109). Compounds **DTT-S6** and **DTT-S8** were selected for further anticancer activity studies on the MCF-7 cell line, since the most antiproliferative activity was observed on the MCF-7 breast cancer cell line.

**Table 1:** IC_50_ values (µM) of compounds **DTT-S1-18** on HUVEC, MCF-7, PC-3, and HT-29 cell lines

Compounds	IC_50_ (µM)			
	HUVEC	MCF-7	PC-3	HT-29
**DTT-S1**	> 100	> 100	> 100	> 100
**DTT-S2**	59.19 ± 0.12	51.66 ± 0.04	94.38 ± 0.09	> 100
**DTT-S3**	> 100	84.53 ± 0.12	97.44 ± 0.09	> 100
**DTT-S4**	> 100	> 100	> 100	> 100
**DTT-S5**	> 100	> 100	> 100	> 100
**DTT-S6**	32.77 ± 0.09	41.61 ± 0.07	62.34 ± 0.08	68.84 ± 0.12
**DTT-S7**	> 100	> 100	> 100	> 100
**DTT-S8**	47.86 ± 0.05	38.87 ± 0.07	76.75 ± 0.13	> 100
**DTT-S9**	61.29 ± 0.06	> 100	> 100	> 100
**DTT-S10**	> 100	> 100	> 100	> 100
**DTT-S11**	> 100	> 100	> 100	73.13 ± 0.23
**DTT-S12**	> 100	> 100	> 100	> 100
**DTT-S13**	92.72 ± 0.14	> 100	> 100	> 100
**DTT-S14**	> 100	> 100	> 100	84.64 ± 0.12
**DTT-S15**	> 100	> 100	> 100	> 100
**DTT-S16**	> 100	> 100	> 100	> 100
**DTT-S17**	> 100	> 100	> 100	> 100
**DTT-S18**	81.87 ± 0.09	> 100	> 100	> 100
**Doxorubicin**	11.4	13.8	15.6	3.16

**Fig. 3:**
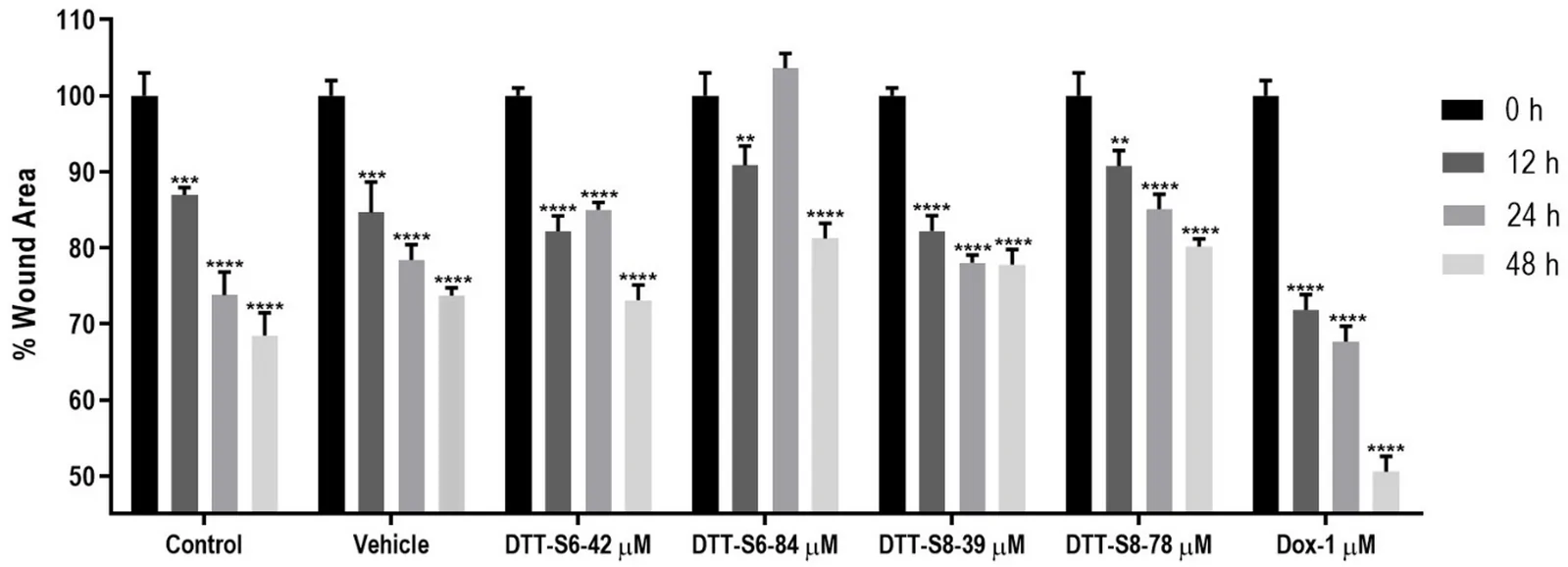
Wound area sizes after 48 h of treatment with **DTT-S6** (42 and 84 µM), **DTT-S8** (39 and 78 µM), and Doxorubucin (1 µM). Data are presented as mean ± SE from three independent experiments (*n* = 3). Statistical significance was determined using one-way ANOVA followed by Dunnett’s multiple comparisons test. Values were compared with the corresponding 0 h time point within each treatment group. **p < 0.02, ***p < 0.0002, ****p < 0.0001

First, the wound healing ability of MCF-7 cells was determined. The wound-healing assay is a simple, inexpensive method used to assess directional cell migration capabilities [38]. Cells were treated with compounds **DTT-S6** and **DTT-S8**, and a linear wound was created in the center of each well to assess cell migration over time. The wound area in the control group decreased by 32% after 48 h. **DTT-S6** and **DTT-S8** did not inhibit cell migration at IC_50_ doses (42 and 39 µM, respectively), and wound area sizes were similar to those of the control group after 48 h, with 27% and 23% reductions, respectively (Fig. 3). At double the IC_50_ doses of **DTT-S6** and **DTT-S8**, wound closure was slightly reduced compared with the control group, with 19% and 21% reductions in wound area after 48 h of treatment, respectively. Doxorubicin was the most effective compound, with a 50% reduction in area size after 48 h. Although **DTT-S6** and **DTT-S8** were identified as the most active compounds in the antiproliferative screening against MCF-7 cells, neither compound produced statistically significant inhibition of cell migration in the wound-healing assay at the tested concentrations compared with the control group. This finding indicates that the growth-inhibitory effects observed in the SRB assay do not necessarily translate into anti-migratory activity. Since cell proliferation and migration are regulated by distinct cellular pathways, compounds may selectively affect one process without significantly influencing the other. Therefore, the biological activity of **DTT-S6** and **DTT-S8** appears to be primarily associated with inhibition of cell growth rather than suppression of migratory behavior. These results suggest that further mechanistic studies are required to elucidate the pathways underlying their antiproliferative activity and to determine whether structural optimization could enhance their anti-migratory potential.

**Fig. 4:**
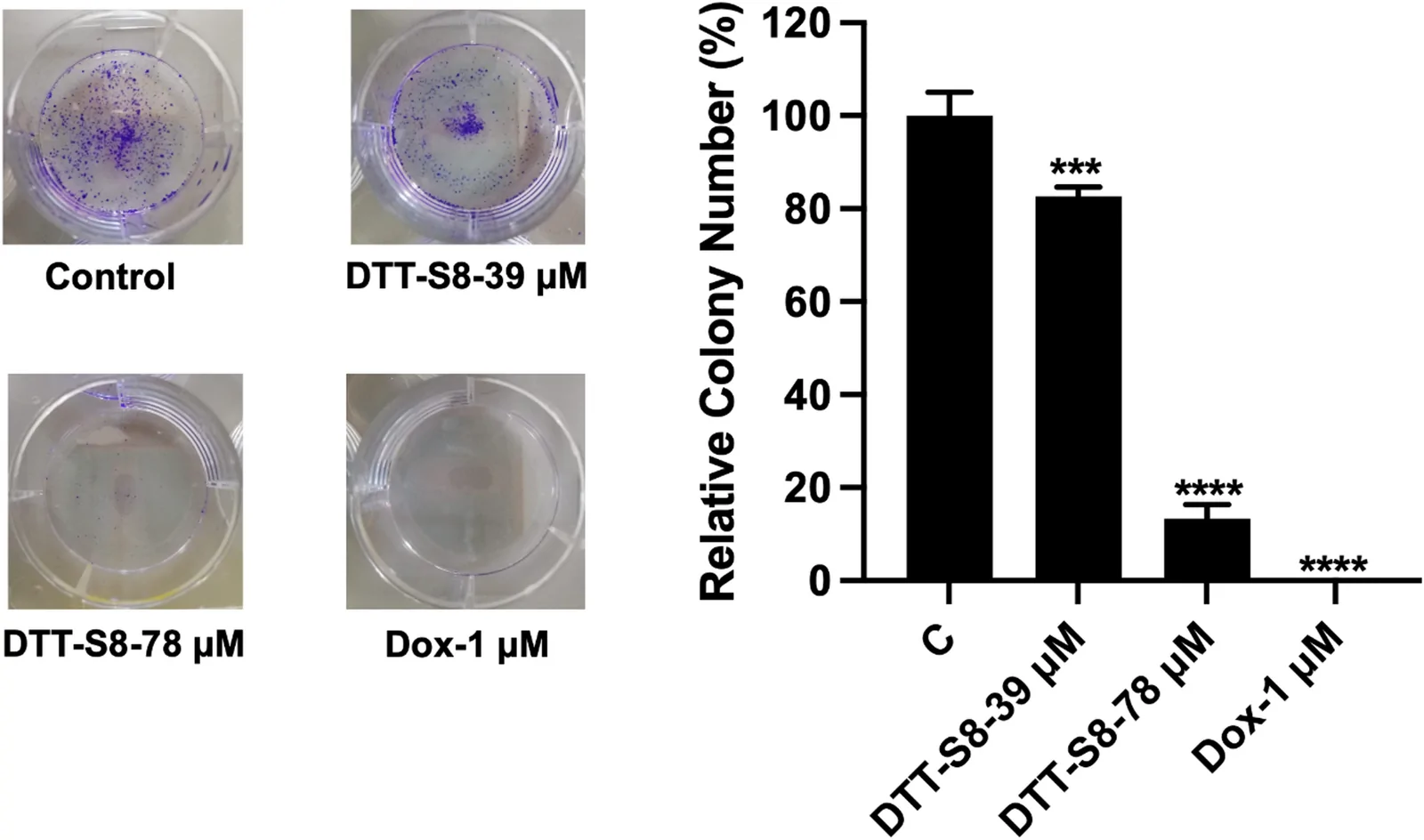
Effect of compound **DTT-S8** on the colony-forming ability of MCF-7 cells. Data are presented as mean ± SE from three independent experiments (*n* = 3). Statistical significance was determined using one-way ANOVA followed by Dunnett’s multiple comparisons test against the control group. C: Control. ***p < 0.0005, ****p < 0.0001

Since the wound areas were not significantly changed with the IC_50_ doses of the compounds **DTT-S6** and **DTT-S8**, long-term survival of MCF-7 cells was evaluated with colony formation experiments with compound **DTT-S8** due to its lower cytotoxicity in healthy endothelial cells. The colony formation assay is essentially useful to detect the ability of a single cell to grow into a colony after treatment with the compounds [39]. The IC_50_ dose of compound **DTT-S8** was unable to inhibit colony formation, however, double the IC_50_ dose of compound **DTT-S8** inhibited colony formation by 81.82% compared to the control group (Fig. 4). Doxorubicin was still the most effective compound for inhibition of colony formation in lower doses which prevented colony formation completely.

Overall, all compounds showed slow release of H_2_S which is favorable for drug candidates. It is essential for candidate drugs to have anticancer activities in lower doses while having low cytotoxicity on healthy cells. Among all compounds, **DTT-S6** and **DTT-S8** showed potential therapeutic activities against cancer cells specifically for MCF-7 breast cancer cells. Even though compounds **DTT-S6** and **DTT-S8** have promising activities, especially compound **DTT-S8**, both compounds were not found as effective as Doxorubicin. More detailed mechanism studies with the synthesis of new derivatives based on compounds **DTT-S6** and **DTT-S8** are required for better understanding of the antiproliferative activities of these compounds.

### Computational studies

#### Homology modeling and validation of the 5-LOX allosteric site

The structural validity of the human 5-LOX allosteric site homology model was established through comprehensive global and local stereochemical evaluations (see Supplementary Text-A; Supplementary Fig. S1). The model showed high fidelity to reference positions (PDB ID: 6NCF) and successfully replicated the binding conformation of acetyl-11-keto-*β*-boswellic acid (AKBA) in redocking studies, confirming its suitability for structure-based virtual screening. However, the modeled catalytic domain lacked sufficient side-chain resolution for Arg596 to reproduce the critical hydrogen bond interaction [40]. Consequently, the inhibitor-bound crystal structure (PDB ID: 6N2W) was utilized for all analyses targeting the active site.

#### Molecular docking

Systematic molecular docking investigations were carried out to predict possible molecular interactions between the newly synthesized DTT derivatives (**DTT-S1-18**) and selected enzymes associated with inflammatory and cancer-related pathways [41–42]. The enzymes selected as targets included COX-1, COX-2, 5-LOX, and iNOS. For 5-LOX, both the catalytic and allosteric binding sites were analyzed separately, given that the enzyme’s allosteric regulatory potential is recognized as a structurally and pharmacologically targetable characteristic [40].

The docking scores, presented in Table 2, were compared with target-specific reference inhibitors to provide a computational framework for interpreting possible target engagement in relation to the observed in vitro antiproliferative activities.

**Table 2:** Glide Extra Precision (XP) docking scores (kcal/mol) of compounds **DTT-S1-18** and the target-specific reference inhibitors against COX-1, COX-2, 5-LOX and iNOS proteins

Compound ID	Glide XP docking score (kcal/mol)				
	COX-1	COX-2	5-LOX		iNOS
			Catalytic site	Allosteric site	
*Test compounds*					
**DTT-S1**	− 5.418	− 6.339	− 4.445	− 4.735	− 2.818
**DTT-S2**	− 8.922	− 7.425	− 4.268	− 4.500	− 2.847
**DTT-S3**	− 8.717	− 7.214	− 4.498	− 4.589	− 2.660
**DTT-S4**	− 7.589	− 8.678	− 5.320	− 3.990	− 3.112
**DTT-S5**	− 7.528	− 7.228	− 5.205	− 3.791	− 2.959
**DTT-S6**	− 7.984	− 8.336	− 5.246	− 5.037	− 3.100
**DTT-S7**	N/A^a^	− 8.493	− 4.123	− 3.971	− 3.393
**DTT-S8**	N/A^a^	− 7.654	− 6.680	− 4.733	− 5.122
**DTT-S9**	N/A^a^	− 8.036	− 5.880	− 5.058	− 3.285
**DTT-S10**	− 7.908	− 8.078	− 5.291	− 5.726	− 3.236
**DTT-S11**	− 6.478	− 7.161	− 4.699	− 5.920	− 4.717
**DTT-S12**	− 9.619	− 7.958	− 5.077	− 5.739	− 4.400
**DTT-S13**	− 7.156	− 7.315	− 4.428	− 5.032	− 3.707
**DTT-S14**	− 7.916	− 9.396	− 4.971	− 5.121	− 4.385
**DTT-S15**	− 9.004	− 10.674	− 5.751	− 4.739	− 4.640
**DTT-S16**	− 7.608	− 9.094	− 5.109	− 5.265	− 4.712
**DTT-S17**	− 7.830	− 9.839	− 5.567	− 5.038	− 5.300
**DTT-S18**	N/A^a^	− 6.330	− 4.624	− 4.123	− 4.595
*Reference inhibitors*					
**COX-1**					
**SC-560**	− 8.994	N/T^b^	N/T^b^	N/T^b^	N/T^b^
**COX-2**					
**RCX**	N/T^b^	− 9.827	N/T^b^	N/T^b^	N/T^b^
***5-LOX*** * Catalytic site*					
**NDGA**	N/T^b^	N/T^b^	− 7.620	N/T^b^	N/T^b^
***5-LOX*** * Allosteric site*					
**AKBA**	N/T^b^	N/T^b^	N/T^b^	− 3.230	N/T^b^
**iNOS**					
**A54**	N/T^b^	N/T^b^	N/T^b^	N/T^b^	− 6.443

^a^N/A (not available) indicates that the docking score was not computed because the Glide workflow did not yield a valid binding pose

^b^N/T: Not tested; compounds other than the known reference inhibitor for each protein were excluded from the assay

Docking results for compound **DTT-S8**, which demonstrated more selective antiproliferative properties within the series (Table 1), supported a hypothesis targeting the COX/LOX axis. Compound **DTT-S8** was found to bind to the COX-2 active site with a binding energy of − 7.654 kcal/mol (Table 2). Although this binding energy was less favorable than that of the reference inhibitor rofecoxib (RCX) at − 9.827 kcal/mol (Table 2), it provides a computational indication of possible COX-2 interaction.

The inability to produce a valid binding pose for COX-1 under the same protocol conditions (Table 2) suggests that **DTT-S8**’s placement in the COX-1 pocket might be structurally restricted. Given that COX-1 inhibition is associated with gastrointestinal and renal adverse effects, a COX-1-avoiding profile is considered a desirable characteristic in an oncological context [43]. COX-2’s pivotal role in processes such as tumor growth, angiogenesis, and immune suppression makes selective COX-2 inhibition a rational strategy for cancer treatment [44].

Furthermore, the docking scores of compound **DTT-S8** for the 5-LOX catalytic site (− 6.680 kcal/mol) and allosteric site (− 4.733 kcal/mol) support a hypothesis of possible COX/LOX-axis involvement rather than demonstrating direct pathway modulation. This predicted interaction profile is consistent with the observed antiproliferative activity of compound **DTT-S8** in MCF-7 cells, although a causal relationship remains to be experimentally established.

Emerging evidence in scientific literature indicates that the simultaneous inhibition of COX-2 and 5-LOX can yield synergistic anti-tumor outcomes in inflammation-associated cancers, frequently demonstrating greater efficacy than single-target inhibition across various tumor models [42]. For instance, Harris et al. reported that the co-administration of COX and LOX inhibitors showcased a more pronounced potential for breast cancer prevention when compared to the application of individual compounds [45]. Additionally, Vaezi et al. identified through several in vitro investigations that the dual inhibition of LOX/COX enzymes effectively led to significant growth inhibition in diverse breast cancer cell lines [46]. These findings suggest that the predicted COX-2/5-LOX interactions of compound **DTT-S8** may provide a plausible mechanistic hypothesis within breast cancer biology, potentially involving the eicosanoid axis.

The docking profile of compound **DTT-S6** indicated a broader-spectrum binding tendency, capable of targeting both COX isoforms simultaneously (COX-1: − 7.984 kcal/mol; COX-2: − 8.336 kcal/mol) (Table 2). While such a pattern holds the potential for a more comprehensive intervention in eicosanoid flow, the relatively strong binding signal to COX-1 necessitates cautious interpretation regarding normal tissue tolerance and therapeutic window [43, 47]. The constitutive expression of COX-1 in gastrointestinal tissue and its role in maintaining tissue integrity explains why COX-1 inhibition is one of the primary determinants of gastrointestinal toxicity associated with non-selective NSAIDs [44]. In this regard, dual COX-inclined profiles like compound **DTT-S6** provide a rational basis for increasing COX-2 orientation through chemical optimization of the DTT scaffold.

Regarding iNOS, the most favorable scores in the series were observed for compound **DTT-S17** (− 5.300 kcal/mol) and compound **DTT-S8** (− 5.122 kcal/mol) (Table 2). However, both showed weaker binding tendencies than the reference inhibitor A54 (− 6.443 kcal/mol) (Table 2). These findings suggest that predicted COX and/or LOX interactions may be more relevant than predicted iNOS interaction for this compound set; however, direct inhibition of these targets was not experimentally confirmed. Given the dual nature of iNOS’s role in cancer, which can be both pro-tumoral and anti-tumoral depending on the tumor type and environment [41], weaker interaction with this target emphasizes the predominant role of other inflammatory mediators. This provided a rational prioritization for moving selected complexes to the next stage of dynamic validation.

Commercial colorimetric COX and iNOS activity kits were initially evaluated experimentally. However, the intrinsic color and strong absorbance of the synthesized compounds caused substantial optical interference, resulting in unreliable assay outputs and preventing accurate determination of enzyme inhibition and IC_50_ values. Therefore, a comprehensive in silico profiling strategy, including validated molecular docking and molecular dynamics simulations, was employed to investigate potential interactions with COX-1, COX-2, and iNOS. These computational results should be considered mechanistic hypotheses rather than direct evidence of enzyme inhibition and warrant future validation using non-colorimetric or orthogonal biochemical assays.

#### MD simulations and MM/GBSA binding free energy calculations

To evaluate whether the binding poses selected by docking were maintained under dynamic conditions and to quantitatively compare binding thermodynamically, 50 ns MD simulations were performed on selected complexes. From these trajectories, average binding free energies (*ΔG*_*bind*_) were calculated using the MM/GBSA approach (Table 3). This computational refinement goes beyond the static nature of docking by partially accounting for binding-pocket flexibility and complex adaptation over time, thereby providing additional support for the predicted binding poses while remaining insufficient to confirm direct enzymatic inhibition [48].

**Table 3:** Average MM/GBSA binding free energies (kcal/mol) of compounds **DTT-S1-18** and target-specific reference inhibitors against COX-1, COX-2, 5-LOX, and iNOS following 50 ns MD simulations

Compound ID	Average MM/GBSA energy^a^ (kcal/mol)				
	COX-1	COX-2	5-LOX		iNOS
			Catalytic site	Allosteric site	
*Test compounds*					
**DTT-S1**	− 60.33 ± 4.86	− 62.71 ± 4.80	− 49.36 ± 5.30	− 48.40 ± 5.16	− 31.94 ± 3.32
**DTT-S2**	− 73.24 ± 4.59	− 65.93 ± 4.01	− 44.87 ± 6.44	− 45.66 ± 7.33	− 42.07 ± 4.32
**DTT-S3**	− 61.88 ± 4.02	− 64.25 ± 5.00	− 41.38 ± 5.17	− 45.28 ± 4.70	− 32.67 ± 3.94
**DTT-S4**	− 70.41 ± 6.21	− 75.98 ± 5.02	− 53.35 ± 5.83	− 47.49 ± 5.99	− 40.40 ± 4.63
**DTT-S5**	− 74.76 ± 6.91	− 77.95 ± 6.01	− 45.88 ± 5.84	− 53.56 ± 4.84	− 39.85 ± 5.12
**DTT-S6**	− 75.31 ± 4.32	− 73.06 ± 4.42	− 59.52 ± 5.50	− 51.34 ± 5.33	− 45.33 ± 5.53
**DTT-S7**	N/A^b^	− 73.51 ± 5.00	− 43.72 ± 6.07	− 53.42 ± 6.60	− 47.76 ± 4.79
**DTT-S8**	N/A^b^	− 82.94 ± 4.91	− 64.42 ± 5.28	− 65.35 ± 4.98	− 44.70 ± 5.07
**DTT-S9**	N/A^b^	− 74.56 ± 4.76	− 53.38 ± 5.94	− 54.88 ± 4.43	− 51.51 ± 4.95
**DTT-S10**	− 74.66 ± 4.61	− 79.05 ± 5.14	− 53.89 ± 5.73	− 54.57 ± 8.06	− 51.14 ± 4.99
**DTT-S11**	− 80.42 ± 6.20	− 68.93 ± 4.54	− 46.39 ± 7.46	− 52.33 ± 7.16	− 43.42 ± 4.58
**DTT-S12**	− 86.25 ± 5.28	− 73.60 ± 4.37	− 52.92 ± 7.83	− 57.69 ± 5.73	− 50.96 ± 4.97
**DTT-S13**	− 58.70 ± 5.33	− 53.79 ± 3.09	− 38.65 ± 6.62	− 35.87 ± 4.87	− 29.79 ± 3.93
**DTT-S14**	− 67.76 ± 4.70	− 71.14 ± 5.66	− 51.07 ± 5.83	− 51.20 ± 4.63	− 37.38 ± 4.14
**DTT-S15**	− 81.06 ± 6.29	− 81.49 ± 6.42	− 69.35 ± 5.62	− 71.88 ± 6.11	− 43.12 ± 5.18
**DTT-S16**	− 72.03 ± 4.98	− 81.68 ± 5.65	− 44.96 ± 6.49	− 55.95 ± 8.80	− 45.20 ± 6.35
**DTT-S17**	− 82.80 ± 5.95	− 81.37 ± 6.47	− 54.96 ± 8.60	− 58.76 ± 7.35	− 43.40 ± 6.26
**DTT-S18**	N/A^b^	− 82.60 ± 6.84	− 60.50 ± 7.97	− 62.17 ± 4.83	− 53.49 ± 5.65
*Reference inhibitors*					
**COX-1**					
**SC-560**	− 66.76 ± 4.45	N/T^c^	N/T^c^	N/T^c^	N/T^c^
**COX-2**					
**RCX**	N/T^c^	− 63.68 ± 4.08	N/T^c^	N/T^c^	N/T^c^
***5-LOX*** * Catalytic site*					
**NDGA**	N/T^c^	N/T^c^	− 34.45 ± 8.33	N/T^c^	N/T^c^
***5-LOX*** * Allosteric site*					
**AKBA**	N/T^c^	N/T^c^	N/T^c^	− 40.98 ± 7.61	N/T^c^
**iNOS**					
**A54**	N/T^c^	N/T^c^	N/T^c^	N/T^c^	− 44.48 ± 3.23

^a^Values represent mean ± standard deviation (SD) (*n* = 3)

^b^N/A: Not available; indicates that average MM/GBSA binding energies could not be computed by Prime due to the absence of a valid Glide XP docking pose

^c^N/T: Not tested; compounds other than the known reference inhibitor for each protein were excluded from the assay

The distribution of *ΔG*_*bind*_ values indicates that the most significant differentiation within the series occurred through COX-2 and 5-LOX interactions. For COX-2, many derivatives exhibited a more favorable energy profile than the reference inhibitor RCX (− 63.68 ± 4.08 kcal/mol) (Table 3). This finding demonstrates that COX-2 binding was not merely an impression based on docking scores but could also be thermodynamically supported under dynamic conditions [49]. In this context, the favorable COX-2 complex predicted for compound **DTT-S8**, with a *ΔG*_*bind*_ of − 82.94 ± 4.91 kcal/mol (Table 3), supports a testable hypothesis that inflammation-related targets may contribute to its antiproliferative profile in MCF-7 cells [50].

The profile of compound **DTT-S8** becomes even more pronounced on the 5-LOX side. Compound **DTT-S8** demonstrated high stability in both the 5-LOX catalytic region (− 64.42 ± 5.28 kcal/mol) and the allosteric region (− 65.35 ± 4.98 kcal/mol), clearly surpassing the energy values of nordihydroguaiaretic acid (NDGA) (− 34.45 ± 8.33 kcal/mol) in the catalytic region and AKBA (− 40.98 ± 7.61 kcal/mol) in the allosteric region (Table 3). This result suggests that the activity of compound **DTT-S8** may not be associated solely with predicted COX-2 interaction but may also involve predicted interactions with both catalytic and allosteric regions of 5-LOX [47]. MM/GBSA analysis results for compound **DTT-S6** confirmed a binding architecture with broad target coverage. Compound **DTT-S6** exhibited strong stability in both COX isoforms, with *ΔG*_*bind*_ values of − 75.31 ± 4.32 kcal/mol for COX-1 and − 73.06 ± 4.42 kcal/mol for COX-2 (Table 3). It also showed significant complex stability with 5-LOX, with values of − 59.52 ± 5.50 kcal/mol in the catalytic region and − 51.34 ± 5.33 kcal/mol in the allosteric region (Table 3). Nevertheless, the limited reflection of these in silico findings in cell viability assays can be attributed to physicochemical and cellular factors such as solubility, cellular permeability, protein binding, and potential off-target effects, which can significantly shape biological responses [51]. Additionally, the low basal activity of COX-2 and 5-LOX in many cell lines, which become prominent only under inflammatory stimuli, might contribute to this inconsistency [52].

The clustering of *ΔG*_*bind*_ values for the iNOS target around reference A54 (− 44.48 ± 3.23 kcal/mol) (Table 3) suggests that iNOS might be a complementary interaction channel rather than a primary determinant for some derivatives in the series. In this scope, compounds **DTT-S6** (− 45.33 ± 5.53 kcal/mol) and **DTT-S8** (− 44.70 ± 5.07 kcal/mol) were in the same energy range as A54, while compounds **DTT-S11** (− 43.42 ± 4.58 kcal/mol), **DTT-S15** (− 43.12 ± 5.18 kcal/mol), and **DTT-S17** (− 43.40 ± 6.26 kcal/mol) showed similar but less favorable values (Table 3).

The predicted balance of COX-2 and 5-LOX interactions may help explain some of the observed phenotypic differences within the series, although additional experimental studies are required to confirm this relationship [53]. In this context, compound **DTT-S8** demonstrated the most consistent thermodynamic multi-targeting signature within the series, aligning with in vitro observations. This was attributed to its ability to form stable complexes with both the binding regions of COX-2 and 5-LOX, while crucially demonstrating selectivity by not forming a stable pose within the COX-1 pocket. Conversely, compound **DTT-S6** exhibited a broad binding profile across the COX/LOX axis, supporting strong bidirectional stability on COX isoforms and possessing significant binding energies in both the catalytic and allosteric regions of 5-LOX. However, due to in silico predictions indicating a risk of COX-1-related side effects and substantial cytotoxicity observed in in vitro HUVEC cell tests, compound **DTT-S6** was classified as a secondary anticancer candidate requiring further structural refinement.

**Fig. 5:**
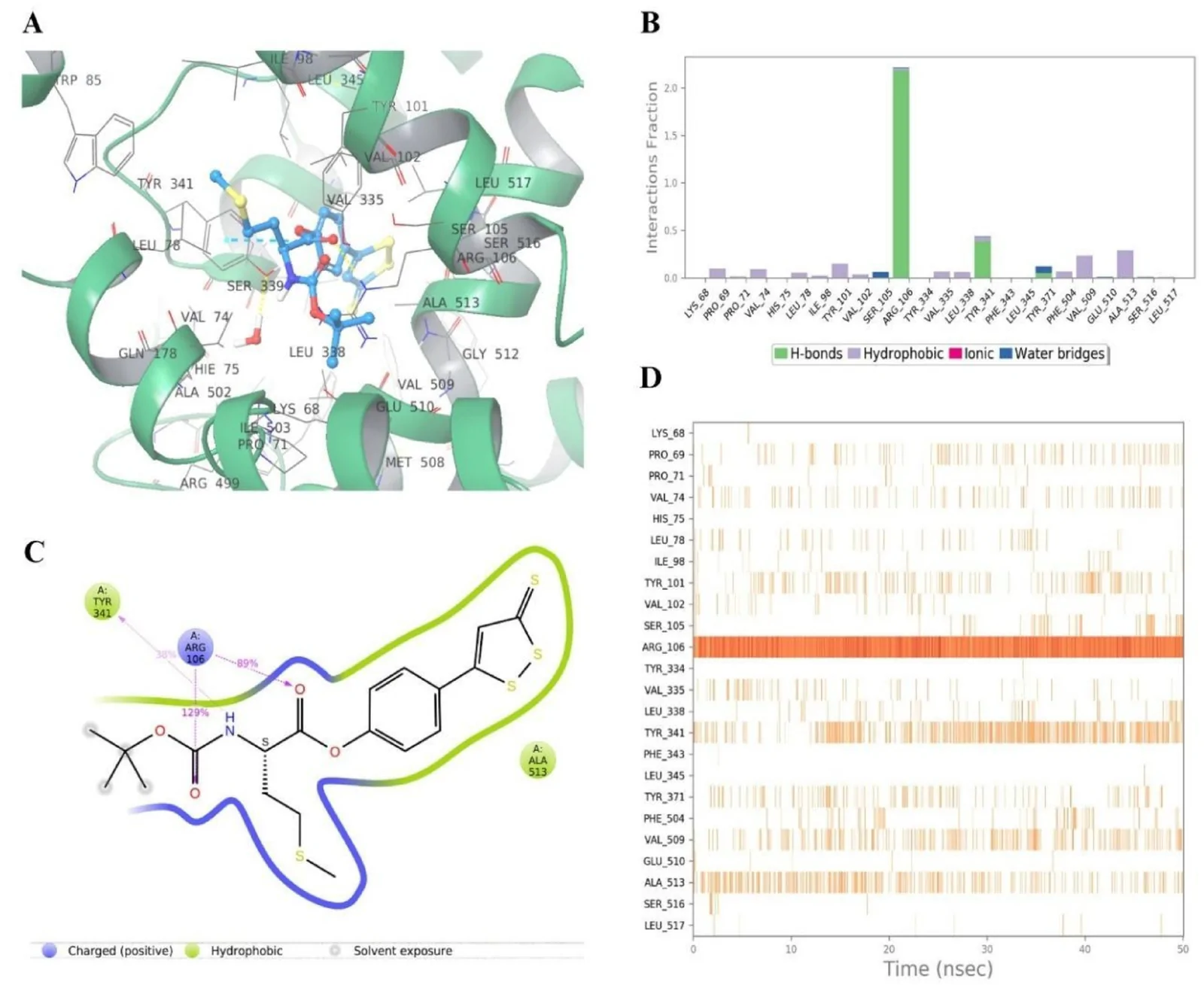
50 ns MD simulations results for compound **DTT-S8** in the COX-2 active site. **A** Glide XP docking pose of compound **DTT-S8** in complex with COX-2. **B** Protein-ligand interaction fractions for key residues throughout the 50 ns trajectory. **C** Two-dimensional (2D) ligand-protein interaction diagram summarizing major contacts (≥ 15% occupancy) between compound **DTT-S8** and COX-2 observed during the simulation. **D** Timeline of residue-specific interactions with compound **DTT-S8** over the 50 ns MD simulation, illustrating the persistence and temporal fluctuations of individual contacts

To elucidate the molecular basis of energetic binding profiles and comparatively assess the stability of the lead compound **DTT-S8** under dynamic conditions against compounds **DTT-S6** and **DTT-S17**, protein-ligand interaction diagrams derived from 50 ns MD simulation trajectories were analyzed. The analysis indicates that compound **DTT-S8** adopts a highly stable binding mode within the COX-2 catalytic channel (Fig. 5A–D). The primary determinant of this stability is the strong electrostatic interactions formed with the Arg106 residue located at the channel entrance, which persisted uninterrupted throughout the simulation (Fig. 5C and D) [49]. This primary binding mechanism, anchoring the ligand to the entrance region via bidirectional hydrogen bonds with 129% and 89% occupancy rates, respectively, is further supported by secondary interactions with Tyr341 (Fig. 5C). Tyr341 restricts the conformational flexibility of the ligand through both hydrogen bond donation (38%) and hydrophobic contacts (Fig. 5B and C), thereby forming a three-point binding motif [54]. Upon examining the ligand’s hydrophobic scaffold within the pocket, van der Waals (vdW) interactions, particularly with Val509 and Ala513 residues, are observed to play a critical role (Fig. 5B and C). The tight hydrophobic packing provided by these residues sterically locks the ligand while minimizing solvent exposure. Consequently, the Arg106-centered polar interaction network and hydrophobic fit within the pocket may provide a structural rationale for the predicted COX-2 binding preference of compound **DTT-S8** [49].

A comparative analysis of compound **DTT-S8** with the reference inhibitor RCX (see Supplementary Text-B; Supplementary Fig. S2A–D) reveals that both ligands share a common hydrophobic locking mechanism facilitated by residues such as Val509 and Ala513. However, a distinct differentiation exists regarding polar anchoring strategies. RCX adopts a side pocket placement, characteristic of selective COX-2 inhibitors and often stabilized by residues like Arg499 (Supplementary Fig. S2C) [55]. In contrast, compound **DTT-S8** prefers to anchor at the channel entrance along the Arg106-Tyr341 axis, rather than entering this specific side pocket (Fig. 5C) [54].

**Fig. 6:**
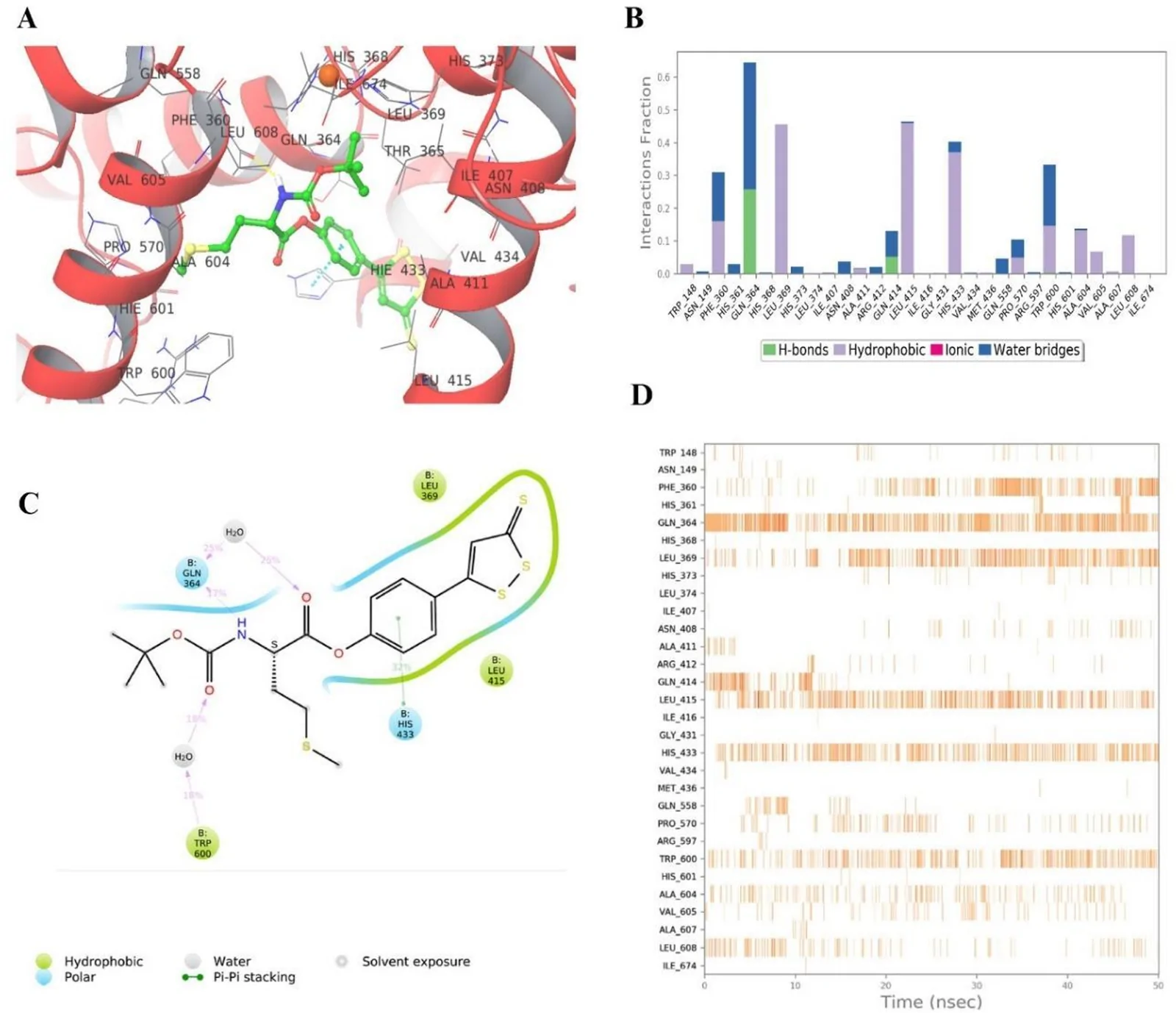
50 ns MD simulations results for compound **DTT-S8** in the 5-LOX active site. **A** Glide XP docking pose of compound **DTT-S8** in a complex with 5-LOX. **B** Protein-ligand interaction fractions for key residues throughout the 50 ns trajectory. **C** 2D ligand-protein interaction diagram summarizing major contacts (≥ 15% occupancy) between compound **DTT-S8** and 5-LOX observed during the simulation. **D** Timeline of residue-specific interactions with compound **DTT-S8** over the 50 ns MD simulation, illustrating the persistence and temporal fluctuations of individual contacts

The antiproliferative profile exhibited by compound **DTT-S8** may be associated not only with H_2_S release but also with its predicted interactions with COX-2 and both catalytic and allosteric regions of 5-LOX. It also stems from its capacity for both catalytic and allosteric modulation within the 5-LOX pathway. Analyses of the 5-LOX catalytic region (Fig. 6A–D) suggest that compound **DTT-S8** maintains a stable predicted binding pose near the catalytic center during the simulation. This is achieved through stable hydrogen bonds with Gln364, located proximally to the iron cofactor, and water-mediated contacts (Fig. 6B and C) [56]. Although some critical interactions observed in reference inhibitors were not detected in these simulations [40], a dense hydrophobic network formed by Phe360, Leu369, Leu415, His433, and Trp600 residues stabilizes the binding pocket (Fig. 6B).

A comparative analysis of compound **DTT-S8** (Fig. 6A–D) with the reference inhibitor NDGA (see Supplementary Text-C; Supplementary Fig. S3A–D) reveals a similar placement profile. Both molecules exhibit common hydrophobic contacts with Phe360, Trp600, and Leu608 residues, which are situated in the deeper regions of the catalytic pocket (Fig. 6B and Supplementary Fig. S3B). However, the polar stabilization mechanisms in the active site demonstrate a distinct strategic divergence. NDGA utilizes His601 as a primary polar anchor point, supported by Arg597, and exhibits an elongated conformation heavily influenced by π-π stacking, effectively filling the orthosteric channel (Supplementary Fig. S3B and C). In contrast, compound **DTT-S8** primarily achieves its stability through a stable network with Gln364 (Fig. 6B and C), independent of Arg597 interactions [57]. Furthermore, compound **DTT-S8** differentiates from the NDGA profile through unique contacts with Leu369 and Leu415 residues, which constitute the enzyme’s plug region [58].

**Fig. 7:**
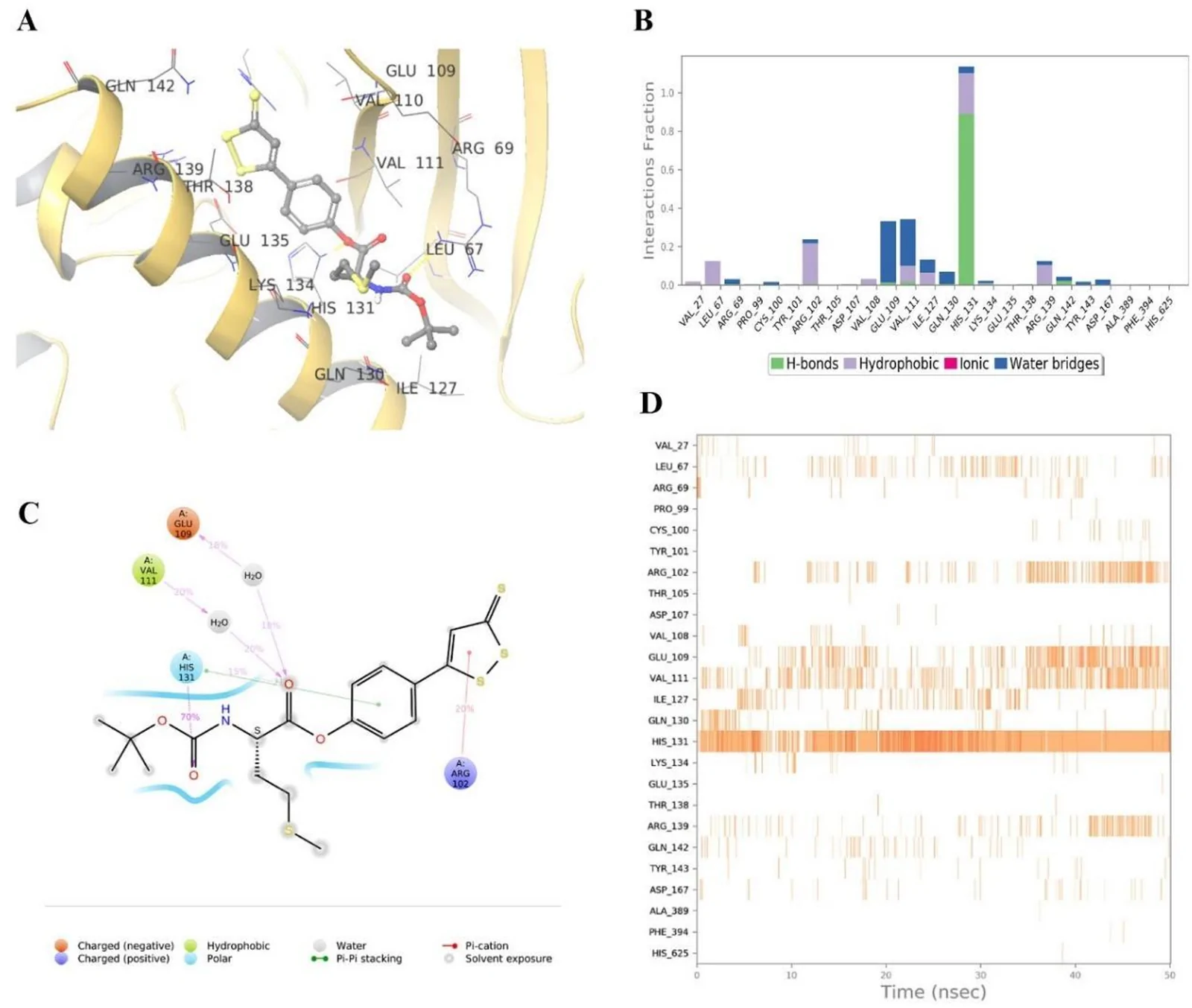
50 ns MD simulations results for compound **DTT-S8** in the 5-LOX allosteric site. **A** Glide XP docking pose of compound **DTT-S8** in a complex with 5-LOX. **B** Protein-ligand interaction fractions for key residues throughout the 50 ns trajectory. **C** 2D ligand-protein interaction diagram summarizing major contacts (≥ 15% occupancy) between compound **DTT-S8** and 5-LOX observed during the simulation. **D** Timeline of residue-specific interactions with compound **DTT-S8** over the 50 ns MD simulation, illustrating the persistence and temporal fluctuations of individual contacts

In the 5-LOX allosteric pocket, compound **DTT-S8** (Fig. 7A–D) closely recapitulates key features of the AKBA binding mode (see Supplementary Text-D; Supplementary Fig. S4A–D). His131 emerges as a dominant interaction hub via a high-occupancy hydrogen bond (70%) and π-π stacking (Fig. 7C), similar to mechanisms reported for AKBA [40]. These anchoring contacts are further supported by water-bridged interactions linking Glu109 and Val111 to the ligand’s polar core, alongside transient π-cation and additional water-mediated contacts involving Arg102 (Fig. 7B–D). Taken together, this interaction fingerprint suggests that compound **DTT-S8** can adopt stable predicted poses at both the catalytic and allosteric regions of 5-LOX, supporting its prioritization for future experimental evaluation of possible COX-2/5-LOX-related activity [59].

When evaluating the binding modes summarized in Supplementary Texts-E-F alongside the dynamic contact profiles in Supplementary Figs. S5 and S6, compound **DTT-S6** appears to exhibit a binding signature with high target-fit, particularly for COX targets, but requires careful consideration regarding selectivity. 50 ns MD simulations reveal that compound **DTT-S6** forms a strong and stable complex within the COX-2 catalytic pocket, characterized by preserved hydrophobic core packing and polar contacts supporting its positioning at the channel entrance (see Supplementary Text-E; Supplementary Fig. S5B–D). The manifestation of similar stabilization within the narrower, apolar active site of COX-1 (see Supplementary Text-F; Supplementary Figures S6A-D) further supports the compound’s tendency for non-selective binding across COX isoforms [49]. Indeed, the more favorable MM/GBSA value obtained for COX-1 compared to SC-560 (Table 3) is a critical finding that thermodynamically reinforces this observation. Furthermore, the placement of compound **DTT-S6** in the deep apolar pocket of COX-1 via the Val348/Tyr384 line, utilizing Arg119 as an electrostatic anchor at the channel entrance (see Supplementary Text-F; Supplementary Fig. S6B–D), indicates a stabilization strategy shared with SC-560’s compact binding (see Supplementary Text-G; Supplementary Fig. S7A–D). However, limited contacts with crucial residues like Ile522 in the selectivity pocket suggest that compound **DTT-S6** does not achieve a selectivity advantage comparable to SC-560 [54]. Consequently, strong COX-1 binding has been interpreted cautiously due to safety risks associated with physiological COX-1 functions [43]. This aligns with the inference that compound **DTT-S8** was preferred as a lead compound, despite compound **DTT-S6**’s potential phenotypic efficacy, owing to compound **DTT-S8**’s more favorable selectivity profile.

Compound **DTT-S6** shows a broader predicted interaction profile beyond COX targets, suggesting its potential for further evaluation against the 5-LOX pathway. This is supported by evidence of sustained stabilization within the 5-LOX catalytic region, achieved through aromatic stacking and a hydrophobic network (see Supplementary Text-H; Supplementary Fig. S8B–D) [60]. Furthermore, the contribution of a polar network, maintained by water-bridges in the allosteric region (see Supplementary Text-I; Supplementary Fig. S9B–D), facilitates stable complex formation, indicating compound **DTT-S6**’s capacity to modulate 5-LOX activity via an allosteric mechanism [40]. In contrast, compound **DTT-S17** is characterized as a multi-targeted candidate, identified by its MM/GBSA-based target profile (Table 3) and its capacity to release H_2_S (Fig. 2). Molecular interaction data also elucidates its dynamic engagement with target pathways (see Supplementary Texts J-L and Supplementary Figs. S10–12). The strategic integration of H_2_S donor moieties into multi-target ligands represents an effective design approach, enabling the simultaneous modulation of diverse signaling pathways to address complex pathophysiological processes [61].

Overall, the integration of 50 ns MD simulations with MM/GBSA binding free-energy calculations provided a theoretical framework for assessing the binding modes and thermodynamic stability of DTT derivatives under simulated physiological conditions. These computational analyses imply that the anticancer potential of these compounds may correlate with predicted interactions involving COX-2 and 5-LOX. Specifically, compound **DTT-S8** was identified as a potentially significant agent, displaying predicted selectivity for COX-2 and stable interactions within both the catalytic and allosteric domains of 5-LOX, indicative of a possible dual-site mechanism. In contrast, the simulation data for compound **DTT-S6** indicated a higher affinity for COX-1, implying possible off-target risks regarding physiological functions. These findings highlight the value of MD simulations in hypothesizing molecular mechanisms that link in vitro anticancer activities with in silico multi-target profiles.

#### Binary QSAR modeling for bioactivity and toxicity predictions

Ligand-based binary QSAR analyses were conducted via the MetaDrug platform to perform a multiparametric evaluation of the drug-likeness properties, as well as the potential anticancer/anti-inflammatory activity profiles and toxicity trends of DTT derivatives (**DTT-S1-18**). Reference inhibitors were included in the dataset to establish a comparative baseline and ensure model reliability. This approach aimed to characterize the antiproliferative phenotype of the series beyond general cytotoxicity, focusing on the suppression of inflammation-related tumor-supporting pathways [62]. Given that chronic inflammation is a key driver of cancer development, progression, and treatment resistance, therapeutic strategies targeting these inflammatory processes represent a vital opportunity in cancer management [42].

All analyzed test compounds met Lipinski’s Rule of 5 (Ro5) criteria (Table 4) [63], indicating that the DTT derivatives’ scaffold is situated within a physicochemical space compatible with oral drug candidates. This finding confirms that the series offers a favorable and optimizable starting point for drug development efforts based on multi-target modulation. When examining the anticancer activity probabilities via TAV scores, a large proportion clustered within the 0.22–0.40 range (Table 4). This distribution suggests a moderate QSAR signal across the series while also indicating that the derivatives possess a unique scaffold not fully overlapping with the chemical space represented in the model’s training set [64]. The Tanimoto Prioritization (TP) values, reflecting prediction reliability, ranged from 32.51 to 44.36% (Table 4). This suggests that the model’s predictions were made through extrapolation rather than strong structural similarity, which could lead to an underestimation of potential activity [65]. Therefore, these in silico data should be interpreted as relative trends within the series rather than absolute values. In this context, cancer TAV values of compounds **DTT-S17** (0.40), **DTT-S8** (0.38), **DTT-S9** (0.38), **DTT-S13** (0.37), and **DTT-S16** (0.36) stood out with relatively high scores, while compound **DTT-S6** (0.31) lagged slightly behind this group (Table 4).

**Table 4:** In silico evaluation of drug-like properties and predicted biological effects, including cancer and inflammation models, for synthesized DTT analogs and reference inhibitors

Compound ID	Property		
	Rule of 5^a^	Cancer (TP^b^)	Inflammation (TP^b^)
*Test compounds*			
**DTT-S1**	OK	0.29 (42.25)	0.52 (31.37)
**DTT-S2**	OK	0.25 (37.19)	0.61 (37.19)
**DTT-S3**	OK	0.29 (36.10)	0.72 (36.10)
**DTT-S4**	OK	0.24 (40.81)	0.78 (38.54)
**DTT-S5**	OK	0.25 (39.50)	0.69 (44.22)
**DTT-S6**	OK	0.31 (37.80)	0.68 (41.63)
**DTT-S7**	OK	0.31 (37.37)	0.70 (40.28)
**DTT-S8**	OK	0.38 (44.36)	0.55 (44.36)
**DTT-S9**	OK	0.38 (41.05)	0.32 (41.05)
**DTT-S10**	OK	0.35 (41.16)	0.63 (50.49)
**DTT-S11**	OK	0.34 (42.35)	0.53 (54.45)
**DTT-S12**	OK	0.27 (42.96)	0.58 (52.79)
**DTT-S13**	OK	0.37 (36.36)	0.53 (30.37)
**DTT-S14**	OK	0.24 (32.51)	0.48 (34.17)
**DTT-S15**	OK	0.33 (40.40)	0.34 (43.69)
**DTT-S16**	OK	0.36 (39.49)	0.33 (39.49)
**DTT-S17**	OK	0.40 (40.91)	0.28 (40.91)
**DTT-S18**	OK	0.22 (42.67)	0.39 (42.67)
*Reference inhibitors*			
**COX-1**			
**SC-560**	OK	0.69 (45.37)	0.85 (66.82)
**COX-2**			
**RCX**	OK	0.30 (43.89)	0.70 (100.00)
**5-LOX Catalytic Site**			
**NDGA**	OK	0.73 (100.00)	0.32 (77.61)
**5-LOX Allosteric Site**			
**AKBA**	OK	0.51 (73.44)	0.46 (81.34)

^a^Compounds complying with Lipinski’s Ro5 are designated as “OK”

^b^TP represents the maximum Tanimoto similarity between the query compound (including metabolites) and the QSAR training set; higher values reflect greater reliability in the model predictions

A comparison of these predictions with in vitro biological data revealed that compounds **DTT-S6** and **DTT-S8** displayed a significant profile. The moderate anticancer activity projected by MetaDrug analysis was found to be consistent with the in vitro cytotoxicity results acquired from the MCF-7 cell line (Tables 2 and 4). Together with their H_2_S-releasing properties and predicted COX-2/5-LOX interaction profiles, this suggests that the observed in vitro activity may result from multiple contributing factors, although a synergistic mechanism has not been experimentally demonstrated [45].

In alignment with the inflammation-cancer hypothesis, the anti-inflammatory activity potentials of the compounds were also investigated, revealing a broad distribution across the series ranging from 0.28 to 0.78 (Table 4). Derivatives **DTT-S4** (0.78), **DTT-S3** (0.72), **DTT-S7** (0.70), and **DTT-S5** (0.69) surpassed the 0.5 threshold, indicating significant probabilities of activity [62]. The lead compound **DTT-S8**, with a TAV score of 0.55, demonstrated a balanced profile for both anticancer and anti-inflammatory activity, while compound **DTT-S17**’s weaker performance in this domain (0.28) further corroborates its biological ineffectiveness. This dual predicted activity profile of compound **DTT-S8** supports its prioritization for future studies assessing whether it can modulate eicosanoid signaling networks that contribute to the tumor microenvironment (TME) [47]. As pivotal regulators of tumor-associated inflammation and cancer growth, eicosanoids serve as a fundamental link between inflammatory processes and tumorigenesis. Therefore, targeting these mediators offers a promising avenue for designing innovative cancer treatments [66].

As indicated in Table 4, the selective COX-1 inhibitor SC-560 (with an anticancer TAV of 0.69) and the selective COX-2 inhibitor RCX (with an anticancer TAV of 0.30) displayed profiles consistent with their established anticancer efficacy reported in scientific literature [67–68]. Similarly, the predicted anticancer scores for the 5-LOX inhibitors NDGA (0.73) and AKBA (0.51) corroborate the known antitumor effects of these compounds [69]. However, the model underestimated the anti-inflammatory activity of potent anti-inflammatory agents like NDGA (anti-inflammatory TAV: 0.32) and AKBA (anti-inflammatory TAV: 0.46). This suggests that the training dataset may not fully capture the diverse anti-inflammatory mechanisms that operate via the LOX pathway [70]. This observation highlights that while in silico predictions offer valuable preliminary insights for DTT derivatives, they should be considered a foundational perspective that necessitates future experimental validation for a comprehensive understanding.

Despite exhibiting similar effectiveness in inhibiting the proliferation of MCF-7 cancer cells (Table 1), the compounds **DTT-S6** and **DTT-S8** show a significant difference in their predicted toxicity profiles, as determined by 26 endpoint toxicity assessments using the MetaDrug platform (see Supplementary Table S1). This disparity is most evident in terms of genotoxicity. Compound **DTT-S6** was predicted to be mutagenic, classified as AMES-positive with a score of 0.87 in the AMES model, whereas compound **DTT-S8** remained within the AMES-negative range with a score of 0.21. This finding indicates that, despite its promising efficacy, compound **DTT-S6** necessitates thorough early-stage validation due to its potential mutagenic risk [71].

Analyzing these toxicity findings alongside molecular binding data strongly supports the selection of compounds based on their balance between selectivity and tolerability. While compound **DTT-S6** binds to both 5-LOX and COX-2, its significant binding to COX-1 raises concerns about potential gastrointestinal and renal side effects [43], which aligns with the negative indicators seen in the MetaDrug toxicity screenings. Conversely, the lack of a valid COX-1 docking pose for compound **DTT-S8** under the applied protocol may indicate a comparatively favorable predicted selectivity profile; nevertheless, this interpretation should be validated by experimental approaches.

### Structure-activity relationships

**Fig. 8:**
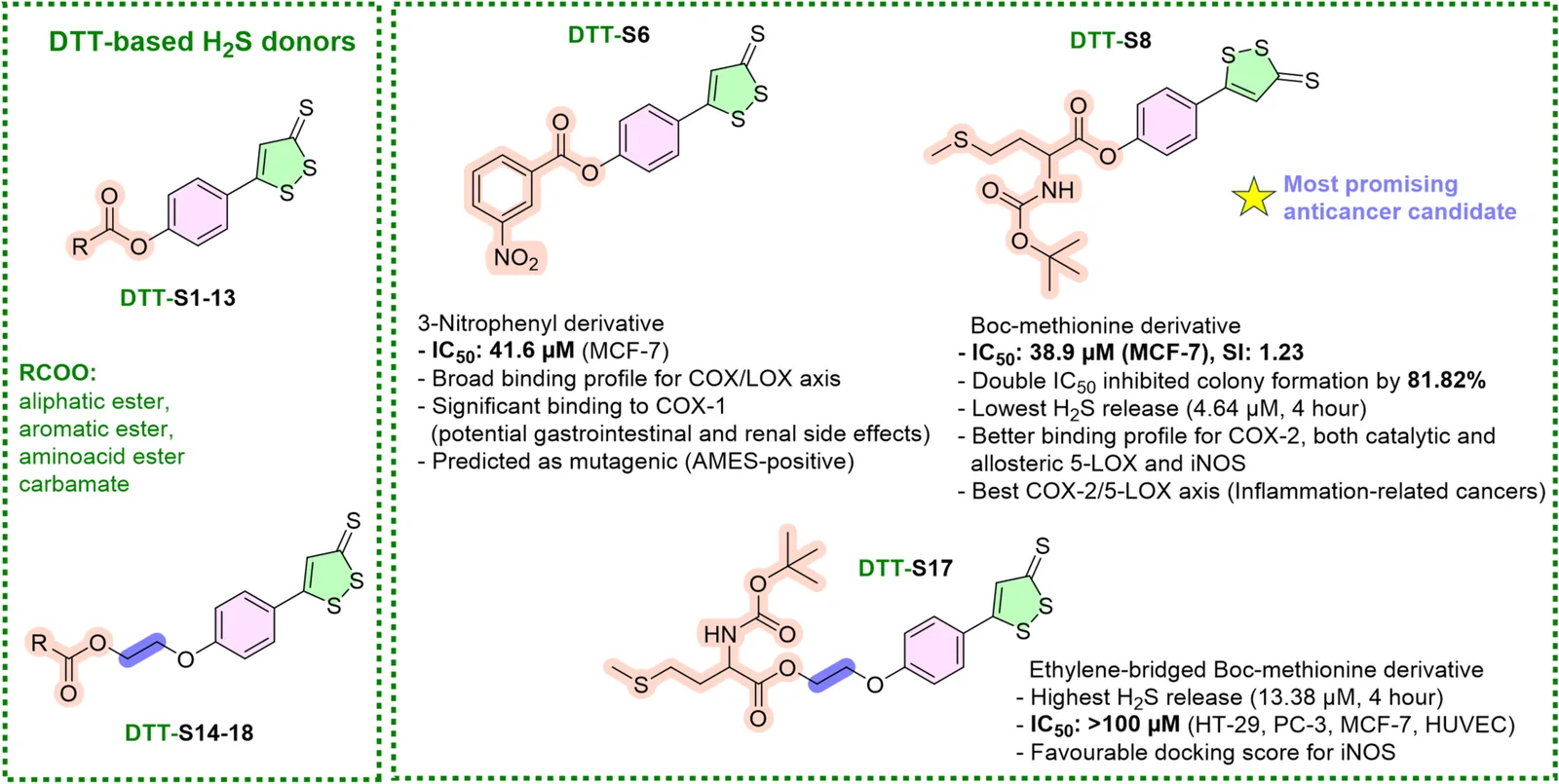
Structure-activity relationships of **DTT-S1-18**

Figure 8 depicts the SAR results for in vitro antiproliferative activity and in silico anti-inflammatory/anticancer activities of newly synthesized DTT-based H_2_S donors (**DTT-S1-18**).

It has been observed that most of the products with the exception of **DTT-S8** showed higher H_2_S release than the standard **ADT-OH**. Of the two Boc-methionine derivatives, **DTT-S8** exhibited the lowest H₂S release (4.64 µM over 4-hour period), whilst **DTT-S17** exhibited the highest H₂S release (13.38 µM). In contrast, compound **DTT-S8** exhibited the best antiproliferative activity on the MCF-7 breast cancer cell line, with the IC_50_ value of 38.87 µM and the selectivity index of 1.23, whereas the ethylene-bridged derivative **DTT-S17** did not significantly inhibit cell growth (> 100 µM) in the MCF-7, HT-29 colon cancer and PC-3 prostate cancer cell lines. Among the aromatic ester derivatives, the IC_50_ value of compound **DTT-S6**, which has nitro group at the 3-position on the phenyl, was determined as 41.61 µM in the MCF-7 cell line. Other substituents on the phenyl ring like as 4-fluoro (**DTT-S4**), 4-nitro (**DTT-S5**), 2-nitro (**DTT-S7**), 3,4,5-trimethoxy (**DTT-S9**) and 4-amido (**DTT-S10-12**) decrease the cytotoxicity. Furthermore, no significant cell cytotoxicities of carbamate derivatives (**DTT-S1**, **DTT-S13**) and ethylene-bridged ester derivatives **(DTT-S14-18)** were observed. In silico studies showed that **DTT-S6** exhibited a broad binding profile for COX isoforms and both catalytic and allosteric sites of 5-LOX. However, the AMES-positive result of this compound and strong binding interaction with COX-1 raise concerns regarding possible mutagenicity and gastrointestinal adverse effects. Similarly, compound **DTT-S8** has also showed the favorable multi-target profile against COX-2, iNOS and both 5-LOX sites. Based on in vitro and in silico results, **DTT-S8** has been identified as the most promising anticancer candidate for breast cancer due to its low IC_50_ value, ability to inhibit colony formation (double IC_50_, 81.82%), and superior in silico performance in the COX-2/5-LOX axis (Fig. 8).

## Conclusion

In conclusion, we successfully synthesized eighteen new DTT-based H_2_S donors (**DTT-S1-18**) containing different functional groups and spectroscopically validated their structures. These compounds demonstrated slow H_2_S release, a profile that better mimics physiological H_2_S dynamics. Cell-based screenings identified compounds **DTT-S6** (3-nitrophenyl derivative) and **DTT-S8** (methionine derivative) as the most active compounds among the synthesized series, exhibiting moderate antiproliferative activity against MCF-7 breast cancer cells. Although the selectivity of the compounds toward cancer cells over healthy HUVEC cells was limited, compound **DTT-S8** displayed a modest preference for MCF-7 cells and inhibited colony formation. Therefore, compound **DTT-S8** may be considered a preliminary lead compound for further structural optimization aimed at improving anticancer potency and cancer-cell selectivity.

Integrated computational analyses suggest that the observed anticancer activity may not be associated solely with H_2_S release and may involve putative interactions with inflammation-related targets, particularly the COX-2/5-LOX axis. However, direct enzymatic inhibition of COX-2, 5-LOX, and iNOS was not experimentally confirmed in this study. Molecular docking, MD simulations, and MM/GBSA calculations predicted stable binding poses of compound **DTT-S8** in the COX-2 catalytic channel and in both catalytic and allosteric regions of 5-LOX. These findings indicate that DTT-based, slow H_2_S-releasing compounds may represent promising scaffolds for the development of multi-target candidates in inflammation-related cancers. Further biochemical and cellular studies are required to determine whether these compounds truly modulate eicosanoid biosynthesis or inflammation-related signaling within the TME.

Future studies will validate the predicted COX-2, 5-LOX, and iNOS interactions using non-colorimetric methods, including LC/MS-based quantification of prostanoids and leukotrienes, Western blot/qPCR analyses, and chemiluminescence-based activity assays. In addition, relevant in vivo models will be used to assess anti-inflammatory and anticancer efficacy and to determine whether these predicted interactions result in functional target inhibition or pathway modulation.

## Experimental section

### Materials and methods

All reagents and solvents were obtained from commercially available sources such as Sigma-Aldrich, Merck, Carlo Erba, Isolab and Tekkim and were used as purchased form without further purification. The melting points were determined using the Electrothermal IA9100 Melting Point Apparatus with a heating rate of 0.5 °C/min. The reaction progression was monitored by thin-layer chromatography (TLC) by using Merck silica gel 60GF_254_ aluminum sheets and spots were viewed under UV light at 254 nm or potassium permanganate stain. Yields refer to isolated compounds after necessary purifications. Infrared spectra of products were recorded on a Perkin Elmer Spectrum 100 FT-IR spectrometer with the ATR technique. LC-MS analyses were performed using a Shimadzu 8040 liquid chromatograph mass spectrometer with ESI ionization technique in positive/negative ionization mode. ^1^H NMR and ^13^C NMR spectra were recorded on Jeol 400/100 MHz and Agilent 600/150 MHz spectrometers with deuterated solvents CDCl_3_ and DMSO-d_6_. The δ values of chemical shifts and J values of coupling constants were expressed in ppm and Hz, respectively. Microwave heating reactions were performed on the CEM Discover SP instrument. The purity of synthesized compounds was found to be > 95% using a Shimadzu Prominence LC-20A semi-preparative HPLC chromatograph equipped with a photodiode array detector. Analyses were conducted using a Shim-pack ODS(H) 250 × 4.6 mm, 5 μm C18 column with 1.0 mL/min flow rate at 45 min. Gradient: 0–5 min 20% B, 5–30 min 20–60% B, 30–35 min 60% B, 35–40 min 60 − 20% B and 40–45 min 20% B (Mobile phase A: methanol and mobile phase B: H_2_O).

### Synthesis of products DTT-S1-18

#### 5-(4-Hydroxyphenyl)-3*H*-1,2-dithiole-3-thione (ADT-OH)

Firstly, **ADT-OMe** (5-(4-methoxyphenyl)-3*H*-1,2-dithiole-3-thione) was synthesized according to a previously reported literature method [27]. Orange crystal; 60% yield; m.p. 106–108 °C; IR (ATR) υ 3083, 3011, 2957, 2932, 2888, 2828, 1600, 1572, 1479, 1256, 1175, 1116, 1022, 817, 690 cm^− 1^; ^1^H NMR (400 MHz, CDCl_3_, ppm): δ 7.61 (dt, J = 8.9, 2.6 Hz, 2 H), 7.38 (s, 1H), 6.97 (dt, J = 8.9, 2.6 Hz, 2 H), 3.87 (s, 3 H). Then, **ADT-OH** was synthesized from **ADT-OMe** according to the literature method [28]. Orange solid; 77% yield; m.p. 189–191 °C (lit. m.p.: 188–189 °C) [72]; IR (ATR) υ 3152, 3050, 3013, 1606, 1586, 1467, 1376, 1180, 829, 669 cm^− 1^; ^1^H NMR (400 MHz, DMSO‑d_6_, ppm): δ 10.47 (s, broad, 1H), 7.75 (dt, J = 8.7, 2.5 Hz, 2 H), 7.66 (s, 1H), 6.85 (dt, J = 8.7, 2.5 Hz, 2 H); ^13^C NMR (100 MHz, DMSO‑d_6_, ppm): 214.9, 174.9, 162.3, 134.0, 129.7, 122.7, 116.9.

#### 4-(3-Thioxo-3*H*-1,2-dithiol-5-yl)phenyl dimethylcarbamate (DTT-S1)

**ADT-OH** (0.44 mmol; 0.1 g) was dissolved in pyridine (2 mL). Dimethylcarbamoyl chloride (0.53 mmol; 0.045 mL; 1.2 eq.) was added dropwise and the mixture was stirred at room temperature for 3 h under nitrogen atmosphere. The reaction was monitored by TLC using hexane:chloroform:acetone/13:10:2 solvent system. On completion of the reaction, the mixture was poured into ice-water (30 mL) and stirred for 15 min. The resulting precipitate was filtered and washed with water. Orange solid; 93 mg; 71% yield; m.p. 163–165 °C; Rf = 0.57 (hexane/chloroform/acetone, 13:10:2); IR (ATR) υ 3077, 3066, 2959, 2919, 2882, 2853, 1712, 1600, 1579, 1487, 1475, 1388, 1158, 1116, 806, 663 cm^− 1^; ^1^H NMR (400 MHz, DMSO‑d_6_, ppm): δ 7.90 (d, J = 8.6 Hz, 2 H), 7.78 (s, 1H), 7.27 (d, J = 8.6 Hz, 2 H), 3.02 (s, 3 H), 2.89 (s, 3 H); ^13^C NMR (100 MHz, DMSO‑d_6_, ppm): 215.9, 173.5, 154.9, 153.9, 136.1, 128.9, 128.6, 123.5, 36.9, 36.7; LC/MS (m/z): C_12_H_11_NO_2_S_3_ requires 297.41 found [M + H]^+^: 298.0; HPLC analysis, t_R_ = 8.61 min, 100% purity.

### General Procedure for Preparation of DTT-S2-7

**ADT-OH** (1.0 mmol; 0.22 g; 1.0 eq.) was dissolved in chloroform (3 mL). The solution was cooled in an ice bath, triethylamine (1.5 mmol; 0.21 mL; 1.5 eq.) and then acyl chloride derivatives (1.5 mmol; 1.5 eq.) were added dropwise. Reaction mixture was stirred at room temperature for 2–6 h. The reaction was monitored by TLC using hexane:ethyl acetate/3:2 solvent system. On completion of the reaction, volatiles were removed on a rotary evaporator. The crude product was purified by column chromatography on silica gel with hexane:ethyl acetate/3:2 to afford the pure products **DTT-S2-7**.

#### 4-(3-Thioxo-3*H*-1,2-dithiol-5-yl)phenyl 3-methylbutanoate (DTT-S2)

Synthesized according to the general procedure for preparation of **DTT-S2-7** using isovaleryl chloride at rt for 6 h. Orange solid; 0.2 g; 65% yield; m.p. 95–99 °C; Rf = 0.7 (hexane/ethyl acetate, 3:2); IR (ATR) υ 3076, 3060, 2967 − 2870, 1752, 1597, 1476, 1405, 1277, 1166, 1088, 821, 688 cm^− 1^; ^1^H NMR (400 MHz, DMSO‑d_6_, ppm): δ 7.95 (d, J = 8.6 Hz, 2 H), 7.80 (s, 1H), 7.28 (d, J = 8.6 Hz, 2 H), 3.66 (broad, 2 H), 2.10 (m, J = 6.7 Hz, 1H), 0.99 (d, J = 6.7 Hz, 6 H); ^13^C NMR (100 MHz, DMSO‑d_6_, ppm): 215.9, 173.1, 171.2, 153.8, 136.2, 129.2, 129.1, 123.6, 42.8, 25.7, 22.5; LC/MS (m/z): C_14_H_14_O_2_S_3_ requires 310.44 found [M + H]^+^: 311.0; HPLC analysis, t_R_ = 9.93 min, 96.46% purity.

#### 4-(3-Thioxo-3*H*-1,2-dithiol-5-yl)phenyl 3,3,3-trifluoropropanoate (DTT-S3)

Synthesized according to the general procedure for preparation of **DTT-S2-7** using 3,3,3-trifluoropropanoyl chloride at rt for 4 h. Orange solid; 0.22 g; 65% yield; m.p. 140–142 °C; Rf = 0.76 (hexane/ethyl acetate, 3:2); IR (ATR) υ 3086, 3062, 2948, 2852, 1763, 1601, 1489, 1412, 1386, 1273, 1186, 1095, 1017, 837, 697 cm^− 1^; ^1^H NMR (400 MHz, DMSO‑d_6_, ppm): δ 7.99 (dt, J = 8.7, 2.4 Hz, 2 H), 7.81 (s, 1H), 7.34 (dt, J = 8.7, 2.4 Hz, 2 H), 4.04 (q, J = 10.7 Hz, 2 H); ^13^C NMR (100 MHz, CDCl_3_, ppm): 215.5, 171.1, 162.1, 152.5, 136.3, 129.9, 128.4, 123.0 (q, J_C−F_ =276.5 Hz), 122.6, 39.8 (q, J_C−F_ =31.7 Hz); LC/MS (m/z): C_12_H_7_F_3_O_2_S_3_ requires 336.36 found [M + H]^+^: 337.0; HPLC analysis, t_R_ = 8.12 min, 100% purity.

#### 4-(3-Thioxo-3*H*-1,2-dithiol-5-yl)phenyl 4-fluorobenzoate (DTT-S4)

Synthesized according to the general procedure for preparation of **DTT-S2-7** using 4-fluorobenzoyl chloride at rt for 4 h. To obtain pure **DTT-S4**, recrystallization with ethyl acetate was applied after column chromatography. Orange solid; 0.1 g; 29% yield; m.p. 172–175 °C; Rf = 0.84 (hexane/ethyl acetate, 3:2); IR (ATR) υ 3113 − 3034, 1734, 1598, 1258, 1221, 1168, 1152, 847, 678 cm^− 1^; ^1^H NMR (400 MHz, DMSO‑d_6_, ppm): δ 8.21 (m, 2 H), 8.00 (d, J = 8.6 Hz, 2 H), 7.82 (s, 1H), 7.47 (d, J = 8.6 Hz, 2 H), 7.43 (t, J = 8.7 Hz, 2 H); ^13^C NMR (100 MHz, DMSO‑d_6_, ppm): 216.1, 173.2, 166.9 (d, J_C−F_ =252.9 Hz), 163.8, 154.1, 136.4, 133.5 (d, J_C−F_ =9.7 Hz), 129.6, 129.3, 125.8, 123.8, 116.8 (d, J_C−F_ =22.2 Hz); LC/MS (m/z): C_16_H_9_FO_2_S_3_ requires 348.42 found [M + H]^+^: 349.0; HPLC analysis, t_R_ = 6.36 min, 95.34% purity.

#### 4-(3-Thioxo-3*H*-1,2-dithiol-5-yl)phenyl 4-nitrobenzoate (DTT-S5)

Synthesized according to the general procedure for preparation of **DTT-S2-7** using 4-nitrobenzoyl chloride at rt for 2 h. The product precipitated in the reaction medium was filtered and purified by recrystallization with ethyl acetate. Bright orange solid; 0.26 g; 70% yield; m.p. 198–199 °C; Rf = 0.74 (hexane/ethyl acetate, 3:2); IR (ATR) υ 3109, 3071, 3049, 1736, 1599, 1521, 1348, 1261, 1166, 1086, 844, 710 cm^− 1^; ^1^H NMR (400 MHz, DMSO‑d_6_, ppm): δ 8.40 (dt, J = 8.9, 2.3 Hz, 2 H), 8.35 (dt, J = 8.9, 2.3 Hz, 2 H), 8.03 (d, J = 8.7 Hz, 2 H), 7.85 (s, 1H), 7.53 (d, J = 8.7 Hz, 2 H); ^13^C NMR (100 MHz, DMSO‑d_6_, ppm): 216.1, 173.2, 163.4, 153.8, 151.2, 136.5, 134.7, 131.9, 129.8, 129.4, 124.6, 123.8; LC/MS (m/z): C_16_H_9_NO_4_S_3_ requires 375.43 found [M + H]^+^: 376.0; HPLC analysis, t_R_ = 5.57 min, 98.49% purity.

#### 4-(3-Thioxo-3*H*-1,2-dithiol-5-yl)phenyl 3-nitrobenzoate (DTT-S6)

Synthesized according to the general procedure for the preparation of **DTT-S2-7** using 3-nitrobenzoyl chloride at rt for 3 h and purified by recrystallization with ethyl acetate. Orange solid; 0.24 g; 65% yield; m.p. 256–258 °C; Rf = 0.6 (hexane/ethyl acetate, 3:2); IR (ATR) υ 3095 − 3042, 1734, 1601, 1525, 1347, 1255, 1172, 1031, 1002, 862, 713, 686 cm^− 1^; ^1^H NMR (400 MHz, DMSO‑d_6_, ppm): δ 8.78 (t, J = 1.9 Hz, 1H), 8.57 (ddd, J = 8.5, 2.5, 0.8 Hz, 1H), 8.52 (dt, J = 7.9, 1.3 Hz, 1H), 8.03 (d, J = 8.7 Hz, 2 H), 7.91 (t, J = 7.9 Hz, 1H), 7.86 (s, 1H), 7.54 (d, J = 8.7 Hz, 2 H); ^13^C NMR (100 MHz, DMSO‑d_6_, ppm): 215.9, 173.1, 163.1, 153.7, 148.4, 136.4, 131.4, 130.8, 129.7, 129.3, 129.0, 124.8, 123.7; LC/MS (m/z): C_16_H_9_NO_4_S_3_ requires 375.43 found [M + H]^+^: 376.0; HPLC analysis, t_R_ = 7.34 min, 97.28% purity.

#### 4-(3-Thioxo-3*H*-1,2-dithiol-5-yl)phenyl 2-nitrobenzoate (DTT-S7)

Synthesized according to the general procedure for preparation of **DTT-S2-7** using 2-nitrobenzoyl chloride at rt for 3 h. Bright orange solid; 0.2 g; 53% yield; m.p. 185–187 °C; Rf = 0.49 (hexane/ethyl acetate, 3:2); IR (ATR) υ 3072, 3044, 1734, 1602, 1575, 1479, 1361, 1255, 1149, 1021, 1004, 827, 668 cm^− 1^; ^1^H NMR (400 MHz, DMSO‑d_6_, ppm): δ 8.19 (dd, J = 7.6,1.5 Hz, 1H), 8.12 (dd, J = 7.6,1.5 Hz, 1H), 8.04 (dt, J = 8.7, 2.4 Hz, 2 H), 7.95 (td, J = 7.6, 1.5 Hz, 1H), 7.90 (td, J = 7.6, 1.5 Hz, 1H), 7.84 (s, 1H), 7.45 (dt, J = 8.7, 2.4 Hz, 2 H); ^13^C NMR (100 MHz, DMSO‑d_6_, ppm): 216.1, 173.0, 163.7, 153.3, 148.1, 136.6, 134.7, 134.1, 130.9, 130.1, 129.6, 125.9, 125.1, 123.3; LC/MS (m/z): C_16_H_9_NO_4_S_3_ requires 375.43 found [M + H]^+^: 376.0; HPLC analysis, t_R_ = 5.66 min, 96.04% purity.

### General Procedure for Preparation of DTT-S8-12

To a stirred solution of **ADT-OH** (1.0 eq.) in DCM, *N*,* N′*-dicyclohexylcarbodiimide (DCC; 1.1 eq.) and 4-dimethylaminopyridine (DMAP; catalytic amount; 0.05 eq.) were subsequently added. Carboxylic acid derivative (1.1 eq.) in DCM was added dropwise, and the reaction mixture was stirred for 2–8 h at room temperature. The reaction was monitored by TLC using hexane:ethyl acetate/3:2 solvent system. On completion of the reaction, 1,3-dicyclohexyl urea was filtered and washed with DCM. The solvent was removed under vacuum. The crude product was purified by column chromatography on silica gel with hexane: ethyl acetate or crystallization with ethyl acetate to afford the pure products **DTT-S8-12.**

#### 4-(3-Thioxo-3*H*-1,2-dithiol-5-yl)phenyl (*tert*-butoxycarbonyl)methioninate (DTT-S8)

Firstly, **Boc-Met-OH** (*N*-(*tert*-butoxycarbonyl)-*L*-methionine) to be used as the starting material was synthesized in 75% yield by the protection reaction of methionine with di-tert-butyl dicarbonate (Boc_2_O) [73]. The second step was performed according to the general procedure for preparation of **DTT-S8-12** using **ADT-OH** (2.0 mmol; 0.45 g), DCC (2.4 mmol; 0.49 g; 1.2 eq.), DMAP (0.05 eq.) and **Boc-Met-OH** (3.2 mmol; 0.79 g; 1.6 eq.) at rt for 8 h. Purification was done with column chromatography using hexane: ethyl acetate (3:2) solvent system. Red solid; 0.54 g; 58% yield; m.p. 95–98 °C; Rf = 0.6 (hexane/ethyl acetate, 3:2); IR (ATR) υ 3368, 3050, 3027, 3008, 2977, 2915, 2856, 1752, 1682, 1598, 1509, 1489, 1366, 1251, 1159, 1025, 857, 668 cm^− 1^; ^1^H NMR (400 MHz, DMSO‑d_6_, ppm): δ 7.97 (d, J = 8.6 Hz, 2 H), 7.79 (s, 1H), 7.58 (d, J = 7.2 Hz, 1H), 7.26 (d, J = 8.6 Hz, 2 H), 4.30 (m, 1H), 2.55 (m, 2 H), 2.04 (s, 3 H and m, 2 H), 1.37 (s, 9 H); ^13^C NMR (100 MHz, DMSO‑d_6_, ppm): 216.0, 173.2, 171.6, 156.3, 153.9, 136.4, 129.5, 129.3, 123.4, 79.2, 53.4, 30.4, 30.1, 28.7, 15.1; LC/MS (m/z): C_19_H_23_NO_4_S_4_ requires 457.65 found [M + H]^+^: 458.0; HPLC analysis, t_R_ = 5.56 min, 100% purity.

#### 4-(3-Thioxo-3*H*-1,2-dithiol-5-yl)phenyl 3,4,5-trimethoxybenzoate (DTT-S9)

Synthesized according to general procedure for the preparation of **DTT-S8-12** using **ADT-OH** (0.44 mmol; 0.1 g), DCC (0.48 mmol; 0.10 g; 1.1 eq.), DMAP (0.05 eq.) and 3,4,5-trimethoxybenzoic acid (0.48 mmol; 0.10 g; 1.1 eq.) at rt for 2 h. Purification was done with column chromatography using hexane:ethyl acetate (2:1) solvent system. Orange solid; 0.125 g; 68% yield; m.p. 167–170 °C; Rf = 0.51 (hexane/ethyl acetate, 2:1); IR (ATR) υ 3100, 3065, 2964, 2940, 2826, 1729, 1652, 1589, 1461, 1414, 1206, 1166, 1026, 861, 669 cm^− 1^; ^1^H NMR (400 MHz, DMSO‑d_6_, ppm): δ 8.02 (d, J = 8.4 Hz, 2 H), 7.86 (s, 1H), 7.48 (d, J = 8.4 Hz, 2 H), 7.41 (s, 2 H), 3.86 (s, 6 H), 3.77 (s, 3 H); ^13^C NMR (100 MHz, DMSO‑d_6_, ppm): 215.9, 173.1, 164.3, 154.1, 153.3, 143.0, 136.3, 129.4, 129.2, 123.9, 123.7, 107.7, 60.7, 56.6; LC/MS (m/z): C_19_H_16_O_5_S_3_ requires 420.51 found [M + H]^+^: 421.0; HPLC analysis, t_R_ = 4.72 min, 98.20% purity.

#### 4-(3-Thioxo-3*H*-1,2-dithiol-5-yl)phenyl 4-(2,2,2-trifluoroacetamido)benzoate (DTT-S10)

Firstly, **4-(2**,**2**,**2-trifluoroacetamido)benzoic acid** to be used as the starting material was synthesized in 78% yield according to the literature method [74]. The second step was performed according to general procedure for preparation of **DTT-S8-12** using **ADT-OH** (0.44 mmol; 0.1 g), DCC (0.48 mmol; 0.1 g; 1.1 eq.), DMAP (0.05 eq.) and 4-(2,2,2-trifluoroacetamido)benzoic acid (0.48 mmol; 0.12 g; 1.1 eq.) at rt for 2 h. Purification was done with recrystallization using ethyl acetate. Red solid; 88 mg; 45% yield; m.p. 215–218 °C; Rf = 0.61 (hexane/ethyl acetate, 3:2); IR (ATR) υ 3324, 3277, 3198, 3127, 3066, 1729, 1705, 1600, 1541, 1264, 1166, 1152, 1065, 851, 687 cm^− 1^; ^1^H NMR (400 MHz, DMSO‑d_6_, ppm): δ 11.61 (s, 1H), 8.17 (d, J = 8.8 Hz, 2 H), 7.99 (d, J = 8.7 Hz, 2 H), 7.90 (d, J = 8.8 Hz, 2 H), 7.82 (s, 1H), 7.47 (d, J = 8.7 Hz, 2 H); ^13^C NMR (100 MHz, DMSO‑d_6_, ppm): 216.1, 173.2, 164.1, 155.4 (q, J_C−F_ =37.5 Hz), 154.1, 142.1, 136.4, 131.7, 129.5, 129.3, 125.7, 123.8, 121.2, 116.1 (q, J_C−F_ =288.7 Hz); LC/MS (m/z): C_18_H_10_F_3_NO_3_S_3_ requires 441.46 found [M + H]^+^: 442.0; HPLC analysis, t_R_ = 5.89 min, 97.42% purity.

#### 4-(3-Thioxo-3*H*-1,2-dithiol-5-yl)phenyl 4-acetamidobenzoate (DTT-S11)

Firstly, **4-acetamidobenzoic acid** to be used as the starting material was synthesized from 4-aminobenzoic acid and acetic acid applying microwave irradiation (200 W, 150 °C, 15 min.) in 51% yield according to the literature method [75]. The second step was performed according to general procedure for preparation of **DTT-S8-12** using **ADT-OH** (0.44 mmol; 0.1 g), DCC (0.48 mmol; 0.1 g; 1.1 eq.), DMAP (0.05 eq.) and 4-acetamidobenzoic acid (0.48 mmol; 87 mg; 1.1 eq.) at rt for 4 h. Purification was done with recrystallization using ethyl acetate. Dark red solid; 86 mg; 50% yield; m.p. 243–246 °C; Rf = 0.51 (hexane/ethyl acetate, 1:3); IR (ATR) υ 3325, 3282, 3189, 3063, 2997, 1720, 1692, 1597, 1525, 1369, 1274, 1071, 1032, 847, 687 cm^− 1^; ^1^H NMR (400 MHz, DMSO‑d_6_, ppm): δ 10.38 (s, 1H), 8.06 (d, J = 8.8 Hz, 2 H), 7.99 (d, J = 8.7 Hz, 2 H), 7.84 (s, 1H), 7.78 (d, J = 8.8 Hz, 2 H), 7.44 (d, J = 8.7 Hz, 2 H), 2.08 (s, 3 H); ^13^C NMR (100 MHz, DMSO‑d_6_, ppm): 216.0, 173.3, 169.7, 164.3, 154.3, 145.1, 136.4, 131.8, 129.4, 129.2, 123.8, 122.9, 118.9, 24.8; LC/MS (m/z): C_18_H_13_NO_3_S_3_ requires 387.49 found [M + H]^+^: 388.0; HPLC analysis, t_R_ = 5.92 min, 97.52% purity.

#### 4-(3-Thioxo-3*H*-1,2-dithiol-5-yl)phenyl 4-propionamidobenzoate (DTT-S12)

Firstly, **4-propionamidobenzoic acid** to be used as the starting material was synthesized from 4-aminobenzoic acid, propionyl chloride and pyridine in 36% yield according to the literature method [76]. The second step was performed according to general procedure for preparation of **DTT-S8-12** using **ADT-OH** (0.44 mmol; 0.1 g), DCC (0.48 mmol; 0.1 g; 1.1 eq.), DMAP (0.05 eq.) and 4-propionamidobenzoic acid (0.48 mmol; 93 mg; 1.1 eq.) at rt for 6 h. Purification was done with column chromatography using hexane:ethyl acetate (2:1) solvent system and then recrystallization with ethyl acetate. Dark red solid; 76 mg; 43% yield; m.p. 196–197 °C; Rf = 0.15 (hexane/ethyl acetate, 2:1); IR (ATR) υ 3318, 3283, 3097 − 3031, 2978, 2936, 1721, 1693, 1593, 1514, 1481, 1270, 1166, 1068, 1020, 844, 689 cm^− 1^; ^1^H NMR (400 MHz, DMSO‑d_6_, ppm): δ 10.28 (s, 1H), 8.06 (d, J = 8.8 Hz, 2 H), 7.98 (d, J = 8.7 Hz, 2 H), 7.82 (s, 1H), 7.78 (d, J = 8.8 Hz, 2 H), 7.44 (d, J = 8.7 Hz, 2 H), 2.36 (q, J = 7.5 Hz, 2 H), 1.07 (t, J = 7.4 Hz, 3 H); ^13^C NMR (100 MHz, DMSO‑d_6_, ppm): 216.1, 173.3, 164.3, 157.1, 154.3, 145.2, 136.4, 131.8, 129.4, 129.2, 123.8, 122.8, 118.9, 33.9, 9.9; LC/MS (m/z): C_19_H_15_NO_3_S_3_ requires 401.51 found [M + H]^+^: 402.0 [; HPLC analysis, t_R_ = 5.92 min, 97.90% purity.

#### 4-(3-Thioxo-3*H*-1,2-dithiol-5-yl)phenyl carbamate (DTT-S13)

**ADT-OH** (1.0 mmol; 0.23 g) was dissolved in DCM (10 mL). Chlorosulfonyl isocyanate (3.0 mmol; 0.26 mL; 3.0 eq.) was added dropwise in an ice bath and the mixture was stirred at 0–5 °C for 5 h under argon atmosphere. The reaction was monitored by TLC using hexane: ethyl acetate/3:2 solvent system. On completion of the reaction, the mixture was poured into ice water (10 mL), stirred for 30 min and extracted with EtOAc (3 × 50 mL). The organic layer was washed with water, dried using anhydrous MgSO_4_ and concentrated under reduced pressure. The residue was subjected to DCM:Et_2_O (6:1) and the resulting solid was filtered and dried. Brown solid; 0.14 g; 53% yield; m.p. 169–171 °C; Rf = 0.48 (hexane/ethyl acetate, 3:2); IR (ATR) υ 3426, 3333, 3265, 3186, 3072, 1697, 1595, 1223, 1172, 831, 677 cm^− 1^; ^1^H NMR (600 MHz, DMSO‑d_6_, ppm): δ 7.89 (d, J = 8.7 Hz, 2 H), 7.77 (s, 1H), 7.25 (d, J = 8.7 Hz, 2 H); ^13^C NMR (150 MHz, DMSO‑d_6_, ppm): 215.95, 173.62, 154.81, 154.56, 136.02, 128.98, 128.31, 123.50; LC/MS (m/z): C_10_H_7_NO_2_S_3_ requires 269.35 found [M + H]^+^: 270.0; HPLC analysis, t_R_ = 5.02 min, 100% purity.

#### 2-(4-(3-Thioxo-3*H*-1,2-dithiol-5-yl)phenoxy)ethyl 2-mercaptoacetate (DTT-S14)

Firstly, compound **ADT-OCH**_**2**_**CH**_**2**_**OH** (5-(4-(2-hydroxyethoxy)phenyl)-3*H*-1,2-dithiole-3-thione) was synthesized from **ADT-OH** and 2-bromoethan-1-ol according to the reported literature method [29]. In the second step, *p*-TsOH (0.1 mmol; 19 mg) and thioglycolic acid (2.0 mmol; 0.14 mL, 2.0 eq.) were added to the stirred solution of **ADT-OCH**_**2**_**CH**_**2**_**OH** (1.0 mmol; 0.27 g) in toluene (15 mL). The mixture was refluxed for 5 h by using a Dean-Stark apparatus. On completion of the reaction, toluene was removed under vacuum. The residue was poured into ice-water (30 mL) and extracted with EtOAc (3 × 30 mL). The organic layers collected were washed with saturated NaHCO_3_ solution (30 mL) and brine (30 mL), dried over anhydrous Na_2_SO_4_. The crude product was obtained after solvent removing in vacuum and purified with column chromatography using hexane:ethyl acetate (3:2) solvent system. Orange solid; 0.11 g; 32% yield; m.p. 90–92 °C; Rf = 0.66 (hexane/ethyl acetate, 3:2); IR (ATR) υ 3110 − 3039, 1740, 1489, 1342, 1251, 1186, 1163, 1056, 879, 696 cm^− 1^; ^1^H NMR (400 MHz, DMSO‑d_6_, ppm): δ 7.84 (d, J = 8.7 Hz, 2 H), 7.74 (s, 1H), 7.07 (d, J = 8.7 Hz, 2 H), 4.42 (m, J = 4.2 Hz, 2 H), 4.29 (m, J = 4.2 Hz, 2 H), 3.78 (s, 2 H); ^13^C NMR (100 MHz, DMSO-d_6_, ppm): 215.2, 174.1, 169.6, 161.9, 134.7, 129.5, 124.4, 115.9, 66.6, 63.8, 40.6; LC/MS (m/z): C_13_H_12_O_3_S_4_ requires 344.48 found [M + H]^+^: 345.0; HPLC analysis, t_R_ = 4.20 min, 95.33% purity.

### General procedure for preparation of DTT-S15-18

**ADT-OCH**_**2**_**CH**_**2**_**Br** was synthesized from **ADT-OH** and 1,2-dibromoethane according to the reported literature method and used as a starting material for **DTT-S15-18** [30]. To a stirred solution of **ADT-OCH**_**2**_**CH**_**2**_**Br** (1.0 mmol; 0.33 g) in acetone (5 mL), K_2_CO_3_ (3.0 mmol; 0.41 g; 3.0 eq.) was added and stirred at room temperature for 15 min. Then, the carboxylic acid derivative (1.1 mmol; 1.1 eq.) was added and the reaction mixture was refluxed at appropriate times (4–20 h). On completion of the reaction, potassium carbonate was filtered and the solvent was removed under vacuum. The residue was poured with water (50 mL) and extracted with EtOAc (3 × 30 mL). The organic layers collected were washed with water (50 mL), dried using anhydrous Na_2_SO_4_, and concentrated under reduced pressure. Purification was done with column chromatography using hexane:ethyl acetate (3:2) solvent system.

#### 2-(4-(3-Thioxo-3*H*-1,2-dithiol-5-yl)phenoxy)ethyl 2-acetamidobenzoate (DTT-S15)

Synthesized according to the general procedure for preparation of **DTT-S15-18** using 2-acetamidobenzoic acid at reflux conditions for 8 h. Orange solid; 0.15 g; 35% yield; m.p. 177–178 °C; Rf = 0.37 (hexane/ethyl acetate, 2:1); IR (ATR) υ 3269, 3065, 3026, 2965, 2929, 2876, 1705, 1682, 1603, 1593, 1487, 1364, 1194, 1163, 1022, 818, 664 cm^− 1^; ^1^H NMR (400 MHz, DMSO‑d_6_, ppm): δ 10.40 (s, 1H), 8.07 (d, J = 8.2 Hz, 1H), 7.86 (d, J = 8.9 Hz, 2 H), 7.83 (dd, J = 6.3, 1.6 Hz, 1H), 7.76 (s, 1H), 7.56 (td, J = 6.3, 1.6 Hz, 1H), 7.17 (t, J = 7.8 Hz, 1H), 7.11 (d, J = 8.9 Hz, 2 H), 4.57 (m, 2 H), 4.42 (m, 2 H), 2.05 (s, 3 H); ^13^C NMR (100 MHz, DMSO‑d_6_, ppm): 215.4, 174.2, 168.9, 167.4, 162.2, 139.9, 134.8, 134.4, 131.0, 129.6, 124.6, 123.8, 122.0, 118.9, 116.2, 66.8, 63.9, 24.9; LC/MS (m/z): C_20_H_17_NO_4_S_3_ requires 431.54 found [M + H]^+^: 432.0; HPLC analysis, t_R_ = 6.16 min, 95.80% purity.

#### 2-(4-(3-Thioxo-3*H*-1,2-dithiol-5-yl)phenoxy)ethyl (*tert*-butoxycarbonyl)alaninate (DTT-S16)

Firstly, **Boc-Ala-OH** (*N*-(*tert*-butoxycarbonyl)-*L*-alanine) to be used as the starting material was synthesized from *L*-alanine and Boc_2_O at similar experimental conditions of methionine protection [73]. Compound **DTT-S16** was synthesized according to the general procedure for preparation of **DTT-S15-18** using **Boc-Ala-OH** at reflux condition for 20 h. Brown solid; 0.20 g; 45% yield; m.p. 123–126 °C; Rf = 0.5 (hexane/ethyl acetate, 3:2); IR (ATR) υ 3387, 3078, 2978, 2925, 2854, 1759, 1690, 1600, 1251, 1180, 1156, 1061, 1014, 827, 662 cm^− 1^; ^1^H NMR (600 MHz, DMSO‑d_6_, ppm): δ 7.85 (d, J = 8.8 Hz, 2 H), 7.74 (s, 1H), 7.27 (d, J = 7.2 Hz, 1H), 7.06 (d, J = 8.8 Hz, 2 H), 4.40 (m, 2 H), 4.26 (m, 2 H), 3.99 (m, J = 7.2 Hz, 1H), 1.32 (s, 9 H), 1.20 (d, J = 7.2 Hz, 3 H); ^13^C NMR (150 MHz, DMSO-d_6_, ppm): 215.4, 174.2, 173.7, 162.2, 155.8, 134.8, 129.6, 124.5, 116.1, 78.7, 66.9, 63.2, 49.6, 28.7, 17.3; LC/MS (m/z): C_19_H_23_NO_5_S_3_ requires 441.58 found [M - H]^−^: 441.0; HPLC analysis, t_R_ = 5.01 min, 95.54% purity.

#### 2-(4-(3-Thioxo-3*H*-1,2-dithiol-5-yl)phenoxy)ethyl (*tert*-butoxycarbonyl)methioninate (DTT-S17)

Synthesized according to the general procedure for preparation of **DTT-S15-18** using **Boc-Met-OH** under reflux condition for 20 h. Orange solid; 0.33 g; 66% yield; m.p. 70–73 °C; Rf = 0.75 (hexane/ethyl acetate, 3:2); IR (ATR) υ 3370, 3073, 2977 − 2917, 1750, 1685, 1601, 1514, 1279, 1175, 1156, 1014, 831, 784 cm^− 1^; ^1^H NMR (600 MHz, DMSO‑d_6_, ppm): δ 7.87 (d, J = 8.2 Hz, 2 H), 7.76 (s, 1H), 7.34 (d, J = 7.5 Hz, 1H), 7.07 (d, J = 8.2 Hz, 2 H), 4.45 (m, 2 H), 4.32 (m, 2 H), 4.10 (q, J = 7.7 Hz, 1H), 2.44 (m, 2 H), 1.96 (s, 3 H), 1.84 (m, 2 H), 1.34 (s, 9 H); ^13^C NMR (150 MHz, DMSO-d_6_, ppm): 215.3, 174.1, 172.9, 162.0, 156.0, 134.7, 129.5, 124.4, 115.9, 78.8, 66.7, 63.3, 52.9, 30.7, 29.9, 28.6, 14.9; LC/MS (m/z): C_21_H_27_NO_5_S_4_ requires 501.69 found [M + H]^+^: 502.0; HPLC analysis, t_R_ = 5.64 min, 100% purity.

#### 2-(4-(3-Thioxo-3*H*-1,2-dithiol-5-yl)phenoxy)ethyl 3,4,5-trimethoxybenzoate (DTT-S18)

Synthesized according to the general procedure for preparation of **DTT-S15-18** using 3,4,5-trimethoxybenzoic acid at reflux condition for 4 h. Orange solid; 0.125 g; 27% yield; m.p. 130–131 °C; Rf = 0.48 (hexane/ethyl acetate, 3:2); IR (ATR) υ 3026, 2994 − 2838, 1700, 1603, 1587, 1481, 1456, 1286, 1191, 1174, 1021, 817, 673 cm^− 1^; ^1^H NMR (600 MHz, DMSO‑d_6_, ppm): δ 7.86 (d, J = 8.9 Hz, 2 H), 7.75 (s, 1H), 7.18 (s, 2 H), 7.12 (d, J = 8.9 Hz, 2 H), 4.59 (t, J = 4.5 Hz, 2 H), 4.44 (t, J = 4.5 Hz, 2 H), 3.77 (s, 6 H), 3.69 (s, 3 H); ^13^C NMR (150 MHz, DMSO-d_6_, ppm): 215.4, 174.2, 165.8, 162.3, 153.3, 142.4, 134.8, 129.6, 125.0, 124.5, 116.2, 107.1, 66.8, 63.8, 60.7, 56.5; LC/MS (m/z): C_21_H_20_O_6_S_3_ requires 464.57 found [M + H]^+^: 465.0; HPLC analysis, t_R_ = 9.45 min, 99.40% purity.

### H_2_S Release of compounds DTT-S1-18

The H_2_S release capacities of compounds **DTT-S1-18** were tested as described by Li et al. with small modifications [77]. Briefly, a reaction mixture containing 20 mM *N*,*N*-dimethyl-*p*-1,4-phenylenediamine sulfate salt (200 µL), and 1% w/v Zn(OAc)_2_ (100 µL) was reacted with 30 mM FeCl_3_ (200 µL). Then, all compounds (100 µM in final) were diluted in 1 mM *L*-cysteine containing phosphate buffer (pH = 7.4). The reaction mixture (500 µL) and the mixture of *L*-cysteine containing compounds (1 mL) were taken into a 24-well plate. The plate was read in a microplate reader (Tecan Infinite^®^ 200 PRO, Switzerland) at 670 nm until a meaningful increase could not be observed.

### Cell culture

HT-29 (human colon cancer), PC-3 (human prostate cancer), MCF-7 (human breast cancer), and HUVEC (human healthy umbilical) cell lines were cultured in Dulbecco’s Modified Eagle Medium (DMEM) supplemented with 10% fetal bovine serum (FBS), 100 U/mL penicillin and 500X Amphotericin B. Cells were incubated at 37 °C in humidified air containing 5% CO_2_. For all the treatment processes, compounds were dissolved in DMSO and maximum DMSO content in wells did not exceed 1.0%.

### Cell cytotoxicity assays

Sulforhodamine B (SRB) was used for the cytotoxicity of the compounds on cancer cell lines (HT-29, PC-3 and MCF-7) and healthy endothelial cell lines (HUVEC). The SRB is the most frequently used method in vitro cytotoxicity test by the National Institute of Health (NIH) for its efficacy in screening studies. The working principle of the test is based on staining the total protein contents of living cells with SRB substance. For the analysis, cells were seeded in 96-well culture plates at a density of 5 × 10^4^ and incubated for 24 h (100 µL). Compounds were applied (1-100 µM) and incubated for 48 h. After incubation, the medium was removed from the wells and live cells were fixed using 10% (m/V) trichloroacetic acid solution (TCA). Then, TCA was removed from the wells, and the wells were washed with distilled water five times. The plate was left for air-drying at room temperature. Fixed cells were stained with 100 µL of 0.04% (m/V) SRB solution. Excess dye was removed, and wells were washed with 1% (V/V) acetic acid four times. SRB bound to the proteins was solubilized with 200 µL of 10 mM (pH: 10.5) Tris-base solution and the plate was incubated on a gyratory shaker for 10 min. The absorbance was determined using a microplate reader (Tecan Infinite^®^ 200 PRO, Switzerland, 540 nm) and IC_50_ values for compounds were calculated. Doxorubicin was used as a reference for positive control.

### Wound healing assay

MCF-7 cells were seeded at a density of 4 × 10^5^ cells per well in 24-well plates and incubated at 37 °C for 24 h to allow attachment. Following incubation, a straight wound was created in the center of each well using a 200 µL pipette tip. The medium was removed, and the wells were washed with 1X PBS. Afterward, wells were treated with compounds at IC_50_ and twice the IC_50_ doses. Images of the cells were captured at four different time intervals (0, 12, 24, and 48 h) using the Launch ImageFocus 4 software integrated with an inverted microscope (Euromex). The wound areas were measured using ImageJ software.

### Clonogenic assay

MCF-7 human breast cancer cells were seeded into 6-well plates at a density of 1000 cells per well and incubated for 24 h to allow attachment. Then, the cells were treated with compounds at IC_50_ and twice the IC_50_ doses for 48 h. The medium was replaced every 2–3 days until sufficient colony formation (approx. 50 cells per colony) was observed. The wells were washed with 1X PBS, and the cells were fixed with 1 mL of 75% methanol and 25% acetic acid solution for one hour at room temperature. Then, the fixative was removed, and 1 mL of 0.05% crystal violet dye was added to each well and incubated for 30 min at room temperature in the dark. The wells were rinsed five times with distilled water, then dried, and the images of the colonies were captured. The colonies were quantified using ImageJ software.

### Statistical analysis

All experiments were performed in at least three independent biological replicates, with each measurement conducted in triplicate unless otherwise stated. Data are expressed as mean ± SE unless otherwise indicated. Statistical analyses were performed using GraphPad Prism version 8. Differences among groups were evaluated using one-way analysis of variance (ANOVA) followed by Dunnett’s multiple comparisons test. A p-value < 0.05 was considered statistically significant. Corresponding significance levels are indicated in the figure legends where applicable.

### Computational studies

#### Protein preparation

The three-dimensional (3D) coordinates for human iNOS (PDB ID: 4NOS), COX-1 (PDB ID: 6Y3C), COX-2 (PDB ID: 5KIR), and 5-LOX (PDB ID: 6N2W) were acquired from the RCSB Protein Data Bank. The protein structures were selected to provide biologically relevant and structurally reliable receptor models, with particular consideration given to species relevance, preservation of catalytic cofactors, availability of experimentally resolved ligand-binding information, and suitability for comparison with target-specific reference inhibitors. To prepare the iNOS structure for docking, the human iNOS crystal structure (PDB ID: 4NOS), which contains the native *S*-ethylisothiourea ligand along with heme and tetrahydrobiopterin (BH_4_) cofactors, was considered. The use of this structure allowed the catalytic environment of the human enzyme to be retained during receptor preparation. The selective inhibitor A54 (2-[(1*R*)-3-amino-1-phenylpropoxy]-4-chlorobenzonitrile) was derived from the *Mus musculus* structure (PDB ID: 2Y37) and structurally aligned into the 4NOS active site using Maestro, ensuring appropriate placement for subsequent analysis. This reference-guided alignment enabled the selective inhibitor-bound conformation to be introduced into the human iNOS catalytic pocket while maintaining the essential cofactors required for enzymatic integrity. In parallel, to overcome the apo state of the human COX-1 structure (PDB ID: 6Y3C), the Celecoxib (CLX)-complexed *Ovis aries* crystal structure (PDB ID: 3KK6) was employed as a structural template. This strategy combined the species relevance of the human COX-1 structure with the ligand-bound information available from the CLX-complexed template. Upon superposition of chain A, the CLX binding mode was mapped onto the 6Y3C structure to conserve the steric and electrostatic properties of the binding pocket. Furthermore, *N*-acetylglucosamine (NAG) moieties conjugated to residues Asn68, Asn144, and Asn410 were retained during preparation. For the COX-2 structure (PDB ID: 5KIR), the native binding environment was preserved by retaining the co-crystallized RCX ligand and associated glycosylation moieties, along with the protoporphyrin IX-cobalt cofactor. The presence of the co-crystallized selective COX-2 inhibitor provided a well-defined reference binding mode, while retention of the cofactor and glycosylation features supported preservation of the structural and catalytic context of the receptor.

In the case of 5-LOX, the catalytic domain within the 6N2W structure was maintained in its functional state by retaining the native inhibitor NDGA and the Fe^2+^ cofactor. This structure was therefore suitable for defining the catalytic binding site in a functionally relevant ligand-bound state. To reconstruct the allosteric region, a homology modeling protocol was executed via the SWISS-MODEL server (https://swissmodel.expasy.org) utilizing the human 5-LOX sequence (UniProt ID: P09917). The high-resolution 7TTK structure (1.98 Å), which shares 98.36% sequence identity with the target, was selected as the template for model generation. Following model generation, the natural inhibitor AKBA was transposed from the 6NCF structure into the allosteric pocket after structural alignment within the Maestro interface. This procedure enabled construction of a structurally consistent allosteric binding model for subsequent docking and MD simulation analyses.

The structural fidelity of the homology model was primarily evaluated through Cα-based RMSD calculations and by benchmarking the re-docked AKBA conformation against experimental coordinates (PDB ID: 6NCF) using the Glide XP algorithm. The 6NCF structure was used as the experimental reference because it contains the resolved AKBA-bound 5-LOX allosteric conformation, allowing direct comparison of the re-docked AKBA pose with the known allosteric binding geometry. Additionally, 2D protein-ligand interaction motifs were analyzed for consistency with previously reported data [40], while global folding quality was quantified using QMEANDisCo and local quality estimate (LQE) metrics [78]. Comprehensive structural validation was subsequently performed using the SAVES 6.1 platform (https://saves.mbi.ucla.edu) by assessing stereochemical quality (Ramachandran plots), non-covalent atomic interactions (ERRAT; threshold ≥ 80%), and 3D-1D compatibility (Verify3D; score ≥ 0.20 for ≥ 80% of residues) [79]. All protein structures were processed using the Protein Preparation Wizard within the Schrödinger suite [80]. Initial preprocessing included the renumbering of residues to align with their respective FASTA sequences, while missing side chains and loops were modeled using the Prime module [81]. Solvent molecules positioned beyond 5.0 Å of the binding pocket were eliminated. Protonation states were generated to simulate physiological conditions (pH 7.4) via PROPKA [82], followed by the optimization of hydrogen-bonding networks and metal coordination geometries. Finally, a restrained energy minimization was performed utilizing the OPLS3e force field [83], continuing until a heavy-atom RMSD convergence threshold of 0.30 Å was reached.

#### Ligand preparation

The 2D structures of the H_2_S-donors (**DTT-S1-18**) were created using the Maestro interface. Positive control compounds were retrieved in SMILES format from the PubChem database (https://pubchem.ncbi.nlm.nih.gov) and integrated into the dataset. Ligand preparation utilized Schrödinger’s LigPrep module to standardize chemical structures. This process involved desaltation, removal of solvent artifacts, and generation of physiologically relevant protonation states at pH 7.0 ± 2.0 via the Epik module [84]. Epik’s role is crucial for predicting pKa values and molecular ionization states, which affect binding predictions. Stereochemical integrity was preserved for defined chiral centers, and up to four stereoisomers were generated for ambiguous centers. Finally, 3D coordinates were computed, and energy minimization was performed using the OPLS3e force field to ensure geometrically optimized ligands for subsequent in silico analyses.

#### Receptor grid generation

Subsequent to protein preparation, receptor grids were precisely defined using the Receptor Grid Generation module within the Schrödinger Suite. This crucial step generates a grid that maps the shape and characteristics of the receptor’s binding site, which is indispensable before commencing ligand docking. For this study, grid boxes were centered on the centroids of the bound ligands to accurately define the binding sites. The Receptor Grid Generation panel allows for specifying various settings, including receptor atom vdW radii and partial charges.

#### Molecular docking

Molecular docking studies were performed to investigate the binding modes of synthesized H_2_S-donor compounds and reference inhibitors against the target proteins. All docking calculations were conducted using the Glide module within the Schrödinger Suite, employing the Glide XP protocol. Glide is a widely used program for predicting protein-ligand binding modes and ranking ligands through virtual screening (VS). The XP protocol involves a more rigorous and less lenient scoring methodology and sampling process compared to other docking modes [85].

For comparative analysis, a panel of reference inhibitors was docked alongside the synthesized compounds. This panel included SC-560, a highly selective COX-1 inhibitor known for its potent inhibition of COX-1-derived PGs, and RCX as a selective inhibitor of COX-2 [86]. NDGA was used, which binds in the catalytic active site of 5-LOX, acting as a redox-type inhibitor lodged within the enzyme’s active site [40]. Additionally, AKBA was used, targeting the allosteric domain of 5-LOX. AKBA is known to wedge between the membrane-binding and catalytic domains, approximately 30 Å from the catalytic iron, thereby influencing regiospecificity and offering a strategy for isoform-specific inhibitor development [40]. Five poses were generated for each ligand, and the pose exhibiting the lowest Glide XP score combined with optimal steric complementarity was selected for detailed interaction analysis.

#### MD simulations and MM/GBSA binding free energy calculations

MD simulations were conducted using Schrödinger’s Desmond module [87] to evaluate the stability and dynamic behavior of the protein-ligand complexes. The top-scoring docking poses were prepared using Desmond’s System Builder, which involved solving the complexes in an orthorhombic box using the TIP3P water model. The systems were neutralized with counter ions and further supplemented with 0.15 M NaCl to accurately mimic physiological ionic strength and conditions.

Simulations employed the OPLS3e force field, which is a comprehensive force field providing broad coverage for drug-like small molecules and proteins, ensuring accurate modeling of bonded and non-bonded interactions. The simulations were run within the isothermal-isobaric (*NPT*) ensemble at 310 K and 1.01325 bar. Temperature was precisely regulated using the Nose-Hoover thermostat, while pressure control was achieved using the Martyna-Tobias-Klein barostat. Production runs were performed for 50 ns in three independent replicates, with 1000 frames recorded per trajectory to ensure sufficient sampling of conformational space.

Following the dynamics phase, binding free energies (*ΔG*_*bind*_) were estimated using the Prime Molecular Mechanics/Generalized Born Surface Area (MM/GBSA) method [48]. This approach, while less computationally demanding than other free energy methods, often provides improved statistical correlations with experimental binding data compared to empirical docking scores. The VSGB 2.1 solvation model [88] was utilized in conjunction with the OPLS3e force field. To account for local induced-fit effects, residues within a 3.0 Å radius of the ligand were treated as flexible during calculations. Calculations were performed on an ensemble of 100 frames extracted at equal intervals from the MD trajectories, providing a more robust estimate of binding affinity by averaging over multiple conformations.

#### Binary QSAR modeling for bioactivity and toxicity predictions

The therapeutic potential and safety profiles of both the synthesized H_2_S donors and reference inhibitors were evaluated using binary QSAR models on the Clarivate Analytics’ MetaDrug platform (https://portal.genego.com). Model reliability was substantiated by TP scores and statistical indicators including sensitivity, specificity, accuracy, and Matthews correlation coefficient (MCC) across training and test sets, alongside drug-likeness assessment via Lipinski’s Ro5 [63]. The “cancer-QSAR” model (N_training_ = 886; N_test_ = 67) employed a decision threshold of 0.5 to categorize activity and demonstrated robust performance metrics defined by a sensitivity of 0.89, specificity of 0.83, accuracy of 0.86, and MCC of 0.72 [89]. Similarly, the “inflammation-model” (N_training_ = 598; N_test_ = 93) utilized the same activity cutoff to achieve a sensitivity of 0.86, specificity of 0.84, accuracy of 0.85, and MCC of 0.69 [62]. Furthermore, toxicological assessments for the analyzed dataset were conducted using a comprehensive suite of 26 different models validated against extensive in vivo and in vitro experimental datasets [71]. These algorithms screened for various safety endpoints including mutagenicity via the Ames test, carcinogenicity in rodent models, genotoxicity, and organ-specific toxicities targeting systems such as the heart, liver, kidneys, lungs, nervous system, and reproductive organs using a probability limit of 0.5. Additionally, regression-based algorithms were utilized to estimate cytotoxicity against the human MCF-7 cell line (pGI_50_) and Maximum Recommended Therapeutic Dose (MRTD) values.

## Supplementary Information

Below is the link to the electronic supplementary material.

Supplementary Material 1

## Data Availability

All supporting data of this study are available within the article and Supplementary Information. The homology model of the human 5-LOX protein, featuring AKBA bound within the allosteric site, has been deposited in the ModelArchive repository and is accessible via the following DOI: https://modelarchive.org/doi/10.5452/ma-o1zxw.
