# Supplementary material for: New 3H-1,2-Dithiole-3-thione derivatives: Design and synthesis, H2S-release profile, in vitro anticancer activity, and in silico multi-target assessment
Source: Mol Divers. 2026 Jul 26;30(6):10387–416. doi: 10.1007/s11030-026-11635-x (PMC13736346; doi:10.1007/s11030-026-11635-x)
Supplement: Supplementary file 1 [file 11030_2026_11635_MOESM1_ESM.docx]

**SUPPLEMENTARY INFORMATION**

**New 3*H*-1,2-Dithiole-3-thione Derivatives: Design and Synthesis, H_2_S-Release Profile, *In Vitro* Anticancer Activity, and *In Silico* Multi-Target Assessment**

Semra ALTUNSOY^1^, Yakup Berkay YILMAZ^2^, Tuğba GÜNGÖR^3,*^, Hazal Nazlıcan ATALAY^2^, Mine ISAOĞLU^4,5^, Serdar DURDAĞI^4,5,6,7^, Mehmet AY^3,*^, Tugba BOYUNEGMEZ TUMER^8,9,*^

^1^Graduate Program of Chemistry, School of Graduate Studies, Canakkale Onsekiz Mart University, Çanakkale, Türkiye

^2^ Graduate Program of Molecular Biology and Genetics, School of Graduate Studies, Canakkale Onsekiz Mart University, Çanakkale, Türkiye

^3^ Natural Products and Drug Research Laboratory, Department of Chemistry, Faculty of Science, Canakkale Onsekiz Mart University, Çanakkale, Türkiye

^4^Computational Biology and Molecular Simulations Laboratory, Department of Biophysics, School of Medicine, Bahçeşehir University, İstanbul, Türkiye

^5^Lab for Innovative Drugs (Lab4IND), Computational Drug Design Center (HİTMER), Bahçeşehir University, İstanbul, Türkiye

^6^Department of Pharmaceutical Chemistry, School of Pharmacy, Bahçeşehir University, Beşiktaş, İstanbul, Türkiye

^7^Quantitative System Biology Lab, Faculty of Medicine, Biruni University, Istanbul, Türkiye

^8^Department of Molecular Biology and Genetics, Faculty of Science, Canakkale Onsekiz Mart University, Çanakkale, Türkiye

^9^Department of Medical Biotechnology, Faculty of Biochemistry, Biophysics and Biotechnology, Jagiellonian University, Kraków, Poland

***Correspondence:** [tgungor@comu.edu.tr](about:blank), [mehmetay06@comu.edu.tr](mailto:mehmetay06@comu.edu.tr), [tumertb@comu.edu.tr](mailto:tumertb@comu.edu.tr)

**Table of Contents**

1. *In silico* studies	p. 2-23
2. FT-IR, ^1^H NMR, ^13^C NMR and MS Spectra of Compounds **DTT-S1-18**	p. 24-62
3. HPLC Purity Control of Compounds **DTT-S1-18**	p. 63-81
4. IC_50_ graphs of compounds **DTT-** **S6** and **DTT-S8** on MCF-7 and HUVEC cells	p. 82

**Supplementary Text A. Homology modeling and validation of the 5-lipoxygenase (5-LOX) allosteric site** The human 5-LOX allosteric site homology model underwent rigorous validation, encompassing backbone structural superposition, redocking-based functional evaluation, and independent stereochemical verification. To confirm the global folding architecture, the model was superimposed onto the experimental template (PDB ID: 6NCF), yielding a Cα-based root-mean-square deviation (RMSD) of 0.287 Å (Figure S1A), which affirmed the preservation of backbone topology and overall tertiary structure (Shin et al., 2017). The competency of the modeled pocket to recognize ligands was assessed by redocking acetyl-11-keto-β-boswellic acid (AKBA) using the Glide XP algorithm. The resulting docking pose showed an RMSD of 0.237 Å relative to the co-crystallized ligand (Figure S1B). This high degree of overlap supports the ability of the docking workflow to reproduce the experimental binding mode and suggests geometric preservation of the allosteric pocket within the homology model (Wu et al., 2024). Analysis of the protein-ligand interaction profile (Figure S1C) revealed a molecular recognition pattern highly congruent with known allosteric binding mechanisms (Gilbert et al., 2020). Key stabilizing interactions included a hydrogen-bonding network connecting AKBA’s 4-carboxylic acid and 14-oxo function to residues Arg102, Val111, and His131. Electrostatic complementarity was further enhanced by contacts between the 3-acetoxy group and the side chains of Glu135 and Arg139. Additionally, the triterpenic scaffold engaged in extensive hydrophobic packing with Tyr143, Val111, Val110, and Val108, consistent with the hydrophobic nature of the interdomain cleft (Gilbert et al., 2020; Liu et al., 2024). The model’s predicted structural quality was assessed using SWISS-MODEL metrics. It yielded a Global Model Quality Estimation (GMQE) of 0.96 and a QMEANDisCo Global score of 0.92 ± 0.05, reflecting a high level of concordance with experimental structures (Waterhouse et al., 2018). Residue-specific analysis via Local Quality Estimate (LQE) showed scores predominantly exceeding 0.7 (Figure S1D), suggesting robust confidence throughout the core regions, with lower scores restricted to flexible terminal loops (Guillon et al., 2024). The structural validity, including stereochemical parameters and atomic contacts, was rigorously assessed using the SAVES v6.1 server. The model achieved an ERRAT quality factor of 91.1%, surpassing the typical threshold for high-quality non-bonded interactions (Colovos & Yeates, 1993). A Verify3D score of 84.82% further corroborated the compatibility between the 3D model and its primary sequence, meeting the criterion that at least 80% of residues should score above 0.2 in the 3D/1D profile (Tenorio et al., 2024). Furthermore, Ramachandran plot analysis (Figure S1E) indicated that 99.8% of residues fell within valid regions (89.2% favored; 10.6% allowed), satisfying stringent stereochemical criteria, as high-quality models typically have over 90% of residues in favored and allowed regions (Wlodawer, 2017).

**Supplementary Text B. Binding Mode and Dynamic Interaction Profiles of Rofecoxib (RCX) within the COX-2 Catalytic Site** RCX consistently adopts the established “canonical coxib pose” within the COX-2 catalytic site (Supplementary Figures S2A-D), a finding supported by previous literature (Limongelli et al., 2010). Its heterocyclic core extends along the channel, with the aryl ring forming tight hydrophobic interactions within a pocket created by Val335, Leu338, Phe504, and Val509 (Figures S2B and S2C). Additional hydrophobic contacts with Ala502, Ile503, and Ala513 further stabilize this binding (Rouzer & Marnett, 2020). The significant COX-2 isoform selectivity of RCX is attributed to its methyl sulfone group, which extends into the COX-2 specific side pocket and forms a stable hydrogen bond with Arg499 (Figures S2B and S2C). This interaction is a known key determinant for the selectivity of methyl sulfonyl-containing inhibitors (Ahmadi et al., 2022). Furthermore, the furanone ring’s carbonyl group forms a secondary hydrogen bond with Ser516, reinforcing binding in the polar region. Time-resolved contact analyses showed the persistence of the Arg499 interaction and hydrophobic contacts with Val509 throughout the simulation, aligning well with known crystallographic structures and supporting the reliability of the simulation protocol (Orlando and Malkowski, 2016).

**Supplementary Text C. Binding mode and dynamic interaction profiles of nordihydroguaiaretic acid (NDGA) within the 5-LOX catalytic site** Our simulations reveal that the inhibitor NDGA adopts an elongated conformation within the 5-LOX catalytic pocket, traversing the orthosteric channel (Supplementary Figures S3A-D), consistent with existing crystallographic data (Gilbert et al., 2020; Liu et al., 2024). The ligand’s two aromatic rings occupy the hydrophobic tunnel, extending from the active region’s entrance to the iron/Ile674 coordination site (Figures S3A and S3B). Interaction analysis showed the catechol ring, near the metal center, forms persistent π-π stacking interactions with Phe360 and Trp600 (Figure S3C). Additional hydrophobic contacts with Ala411, Ala604, Ala607, and Leu608 stabilize the ligand within the lipophilic pocket. Polar interactions are centered around the catalytic iron, where NDGA’s hydroxyl groups form a direct hydrogen bond with His601, serving as a primary polar anchor, and establish water-mediated hydrogen bonds with Gln364 and Thr365 (Figures S3B and S3C). This interaction pattern aligns with NDGA’s redox inhibition mechanism, which involves reorganizing histidine residues in the iron coordination sphere and blocking access to the iron’s coordination site (Gilbert et al., 2020). Time-dependent contact analyses confirmed sustained, high-occupancy interactions with Trp600 and His601, alongside intermittent contacts with Arg597 (Figure S3D). These findings are consistent with the reported binding mode of NDGA within 5-LOX and highlight the Phe360/Trp600-His601 axis and the lipophilic subpocket around Leu369, Leu608, and Ile674 as useful regions for future inhibitor design (Liu et al., 2024).

**Supplementary Text D. Binding mode and dynamic interaction profiles of AKBA within the 5-LOX allosteric site** Our analysis confirms that AKBA (Supplementary Figures S4A-D) binds within the interdomain cleft of 5-LOX, engaging in hydrophobic interactions with Val108 and Tyr143, and forming strong polar contacts with Arg69, Arg102, and Arg113 (Gilbert et al., 2020). Simulation data highlight Arg102 as a critical determinant, maintaining continuous direct hydrogen bonds and ionic contacts. Key residues such as Val111, Glu109, His131 and Gln142 and Asp167 also contribute to the complex’s stability (Figures S4B-D) (Liu et al., 2024). Crucially, AKBA’s binding within this allosteric groove disrupts the Arg102-Asp167 salt bridge and Trp103-Arg166 π-cation interaction, leading to α2 helix rearrangements (Rakonjac Ryge et al., 2014). These reported structural changes may alter substrate access to the catalytic center, providing a useful computational reference for evaluating novel derivatives predicted to interact with this allosteric pocket.

**Supplementary Text E. Binding mode and dynamic interaction profiles of DTT-S6 within the cyclooxygenase-2 (COX-2) catalytic site.** Compound **DTT-S6** binds within the COX-2 catalytic pocket primarily through hydrophobic interactions, embedding among residues such as Leu78, Val102, Val335, Tyr341, and Val509 (Supplementary Figures S5A-D). Arg106 is essential for stabilizing ligand-protein complexes in COX-2, likely contributing to **DTT-S6**’s binding stability at the channel entrance (Rouzer & Marnett, 2020). **DTT-S6** exhibits a distinct binding profile compared to **DTT-S8**. While **DTT-S8** directly interacts with Tyr341 via the amide group (Figure 5C), **DTT-S6** forms multiple hydrogen bonds through its nitro group and water-mediated polar contacts with Glu510 via its carbonyl group (Figure S5C). Energetic analyses indicate **DTT-S8**’s three-point polar mechanism, involving Arg106 and Tyr341, is thermodynamically more favorable than **DTT-S6**’s nitro/hydrophobic-centric mode (Table 3). Both **DTT-S6** and **DTT-S8** interact with Tyr341 and Val509. COX-2 isoform selectivity is significantly influenced by the presence of Val509 in COX-2 (instead of Ile522 in COX-1), which enlarges the active site (Limongelli et al., 2010). This collective interaction profile suggests a stable predicted binding pose of **DTT-S6** within the COX-2 catalytic pocket.

**Supplementary Text F. Binding mode and dynamic interaction profiles of DTT-S6 within the cyclooxygenase-1 (COX-1) catalytic site** Compound **DTT-S6** distinguishes itself from **DTT-S8** by deeply embedding within the narrow, apolar catalytic pocket of the COX-1 isoform (Supplementary Figures 6A-D), demonstrating high structural complementarity. This predicted interaction may indicate a potential COX-1-related safety concern, although this requires experimental confirmation (Faki & Er, 2021). Molecular dynamics (MD) simulations show that **DTT-S6** maintains consistent hydrophobic contacts with residues Val115, Val348, Tyr354, and Ala526 within the COX-1 cavity, effectively occupying the hydrophobic space (Figures S6B and S6C). Additionally, its nitrophenyl moiety engages in π-π stacking interactions with Tyr354, which helps to optimally orient the aromatic core within the amphiphilic subpockets (Nguyen et al., 2021). Furthermore, the ester carbonyl group of **DTT-S6** forms stable hydrogen bonds, water-mediated polar contacts, and salt bridge interactions, including π-cation contributions via Arg119 (Figures S6B-D). These interactions align with the gatekeeper function of the Arg119-Glu523 axis, crucial for the hydrogen bond network at the COX-1 active site entrance (Limongelli et al., 2010). The persistence of these contacts, particularly involving Arg119, Tyr354, and Ala526 throughout the 50 ns simulations (Figure S6D), suggests a stable predicted binding pose for **DTT-S6** under the applied simulation conditions. Such sustained interactions in MD simulations are often correlated with ligand binding stability in COX-1 (Rouzer & Marnett, 2020). This predicted structural stability is consistent with the calculated favorable MM/GBSA binding free energy of -75.31 ± 4.32 kcal/mol (Table 3).

**Supplementary Text G. Binding mode and dynamic interaction profiles of SC-560 within the COX-1 catalytic site** SC-560 adopts a compact, stable pose within the COX-1 catalytic pocket throughout 50 ns MD simulations (Supplementary Figures S7A-D), anchored by a π-cation interaction between its pyrazole ring and Arg119, enabling embedding of aromatic moieties into a hydrophobic subpocket lined by Val348, Tyr384, Trp386, and Ile522 (Rouzer & Marnett, 2020). The trifluoromethyl and chlorophenyl groups occupy a narrow hydrophobic clamp formed by Ile522, Met521, and Phe517 (Figures S7B and S7C). In COX-2, homologous smaller residues like Val509 hinder tight packing, underpinning SC-560’s isoform selectivity (Limongelli et al., 2010). Persistent hydrophobic contacts with Tyr384 and Ile522 lock the ligand (Figures S7C and S7D), promoting prolonged residence time. These observations are consistent with the reported high affinity and approximately 1000-fold selectivity of SC-560 for COX-1 over COX-2 (Smith et al., 1998) and provide a reference computational framework for analyzing the predicted binding profiles of DTT derivatives.

**Supplementary Text H. Binding mode and dynamic interaction profiles of DTT-S6 within the 5-LOX catalytic site** Analysis of the 5-LOX catalytic region suggests that **DTT-S6** (Supplementary Figures S8A-D) may stabilize its predicted binding pose primarily through π-π stacking interactions within the active site (Md Idris et al., 2022). The nitrophenyl ring of **DTT-S6** forms strong π-π stacking contacts with Phe360 and Trp600, while its 1,2-dithiole-3-thione (DTT) scaffold interacts with His433 (Figures S8B and S8C). These interactions provide strong geometric and electronic complementarity in the catalytic pocket’s aromatic sub-region. An extensive hydrophobic network, including residues such as Leu369, Leu415, His601, and Ala604 (Figure S8B), further supports ligand stabilization. Unlike NDGA (Figure S3B), which forms a stable hydrogen bond with Arg597, **DTT-S6** shows only limited, water-mediated, and weak hydrophobic contacts with this residue (Figures S8B and S8D). This lack of robust polar anchoring at Arg597 may limit the predicted stability of **DTT-S6** within the catalytic site (Gilbert et al., 2020). The calculated MM/GBSA binding free energy for **DTT-S6** in the 5-LOX catalytic pocket is consistent with these findings (Table 3), suggesting a moderate predicted interaction profile rather than confirming functional 5-LOX inhibition.

**Supplementary Text I. Binding mode and dynamic interaction profiles of DTT-S6 within the 5-LOX allosteric site** Compound **DTT-S6** occupies an organized, albeit solvent-exposed, binding geometry within the 5-LOX allosteric site (Supplementary Figures S9A-D). Its stabilization primarily stems from hydrogen bonds and water-mediated polar contacts involving residues Glu109, Val111, Arg113, and His131. Val111 and His131 are identified as crucial anchoring residues (Gilbert et al., 2020) (Figures S9B and S9C). Hydrophobic interactions with Arg102, Val110, Leu112, Arg139, and Ala389 are intermittent due to the inherent flexibility of the allosteric pocket but still contribute to the overall binding stability (Figures S9B and S9D) (Liu et al., 2024). MD simulations show a continuous polar interaction network, in contrast to the sporadic nature of hydrophobic contacts (Figures S9C and S9D). Despite this, **DTT-S6** exhibits a binding free energy of -51.34 ± 5.33 kcal/mol, which is less favorable than the -65.35 ± 5.28 kcal/mol observed for the lead molecule **DTT-S8** (Table 3). This less favorable predicted binding profile for **DTT-S6** may be related to its inability to form a stable hydrogen bond with the critical His131 residue, suggesting thermodynamically weaker allosteric interactions compared to **DTT-S8**.

**Supplementary Text J. Binding mode and dynamic interaction profiles of DTT-S17 within the COX-2 catalytic site** Despite a favorable predicted binding-energy profile (Table 3), **DTT-S17** showed no cytotoxicity across the evaluated cell lines (Table 1). To understand this, its interaction profile within the COX-2 binding pocket was investigated (Supplementary Figures S10A-D). **DTT-S17** forms a complex interaction network, more extensive than that of the lead molecule **DTT-S8** (Figures 5B-D). Its aromatic core establishes persistent hydrophobic contacts with residues such as Val102, Val335, Phe343, and Leu345 (Figures S10B-D). Additionally, long-lived interactions with Tyr341 stabilize binding within the catalytic domain (Figure S10D). Regarding polar interactions, Arg106 significantly stabilizes **DTT-S17** via water-mediated hydrogen bonds, partial ionic contacts, and π-cation interactions (Figures S10B and S10C). The critical role of arginine residues at the cyclooxygenase channel entrance in ligand recognition by COX-2 is well documented (Rouzer & Marnett, 2020). Additionally, the terminal ester carbonyl of **DTT-S17**’s alkyl thioester engages in recurrent polar interactions with Tyr101, with transient contacts to Ser105 (Figure S10C). This combination of hydrophobic and polar interactions explains **DTT-S17**’s favorable MM/GBSA binding free energy (Table 3).

**Supplementary Text K. Binding mode and dynamic interaction profiles of DTT-S17 within the 5-LOX catalytic site** Compound **DTT-S17** primarily stabilizes within the 5-LOX catalytic region through compact hydrophobic interactions (Supplementary Figures S11A-D). MD simulations show the **DTT-S17**’s DTT ring forms sustained contacts with Leu608, a known inhibitor-binding residue, while its phenyl moiety interacts with Ile407 (Figures S11B and S11C). This binding mode aligns with the hydrophobic nature of the 5-LOX catalytic site (Liu et al., 2024). A persistent hydrogen bond with Gln364 characterizes **DTT-S17**’s polar interaction profile (Figures S11C and S11D). Notably, **DTT-S17** does not engage Arg597 (Figure S11B), distinguishing it from NDGA’s binding (Figure S3B), where this interaction is crucial for inhibitory potency. Furthermore, **DTT-S17** exhibits weak interactions with the iron-coordinating residues His368 and His373, suggesting a limited direct influence on the catalytic metal center (Gilbert et al., 2020). These observations are consistent with its moderately favorable predicted MM/GBSA binding free energy for the catalytic region (Table 3).

**Supplementary Text L. Binding mode and dynamic interaction profiles of DTT-S17 within the 5-LOX allosteric site** Compound **DTT-S17** demonstrates a thermodynamically favorable and stable interaction within the 5-LOX allosteric site (Supplementary Figures S12A-D), spreading across a large surface area in a conformation similar to the known allosteric inhibitor AKBA (Figures S4B-D). Its aromatic core is anchored via π-cation interactions with Arg102 (Figures S12B and S12C), a critical anchoring point also used by AKBA (Gilbert et al., 2020). **DTT-S17** further establishes a multi-focal interaction network, including water-mediated contacts with His131 and Thr138, and interactions with commonly targeted residues like Arg69 and Glu109, similar to AKBA (Liu et al., 2024). This predicted binding profile suggests that **DTT-S17** may be a useful candidate for further experimental evaluation of possible allosteric 5-LOX modulation.

**Figure S1.** Structural validation and quality assessment of the 5-LOX allosteric site homology model. (A) Superposition of the homology model (orange) and the reference structure (6NCF, blue) based on Cα alignment. (B) Overlay of Glide XP docking poses for AKBA in the model (orange) versus the 6NCF structure (blue). (C) 2D protein-ligand interaction diagram of AKBA within the allosteric site. (D) Residue-wise LQE profile. (E) Ramachandran plot showing the distribution of backbone dihedral angles (φ-ψ).

**Figure S2.** 50 ns molecular dynamics (MD) simulation results for **RCX** in the COX-2 active site. (A) Glide XP docking pose of RCX in complex with COX-2. (B) Protein-ligand interaction fractions for key residues throughout the 50 ns trajectory. (C) 2D ligand-protein interaction diagram summarizing major contacts (≥ 15% occupancy) between RCX and COX-2 observed during the simulation. (D) Timeline of residue-specific interactions with RCX over the 50 ns MD simulation, illustrating the persistence and temporal fluctuations of individual contacts.

**Figure S3.** 50 ns MD simulation results for **NDGA** in the 5-LOX active site. (A) Glide XP docking pose of NDGA in complex with 5-LOX. (B) Protein-ligand interaction fractions for key residues throughout the 50 ns trajectory. (C) 2D ligand-protein interaction diagram summarizing major contacts (≥ 15% occupancy) between NDGA and 5-LOX observed during the simulation. (D) Timeline of residue-specific interactions with NDGA over the 50 ns MD simulation, illustrating the persistence and temporal fluctuations of individual contacts.

**Figure S4.** 50 ns MD simulation results for **AKBA** in the 5-LOX allosteric site. (A) Glide XP docking pose of AKBA in complex with 5-LOX. (B) Protein-ligand interaction fractions for key residues throughout the 50 ns trajectory. (C) 2D ligand-protein interaction diagram summarizing major contacts (≥ 15% occupancy) between AKBA and 5-LOX observed during the simulation. (D) Timeline of residue-specific interactions with AKBA over the 50 ns MD simulation, illustrating the persistence and temporal fluctuations of individual contacts.

**Figure S5.** 50 ns MD simulation results for **DTT-S6** in the COX-2 active site. (A) Glide XP docking pose of **DTT-S6** in complex with COX-2. (B) Protein-ligand interaction fractions for key residues throughout the 50 ns trajectory. (C) 2D ligand-protein interaction diagram summarizing major contacts (≥ 15% occupancy) between **DTT-S6** and COX-2 observed during the simulation. (D) Timeline of residue-specific interactions with **DTT-S6** over the 50 ns MD simulation, illustrating the persistence and temporal fluctuations of individual contacts.

**Figure S6.** 50 ns MD simulation results for **DTT-S6** in the COX-1 active site. (A) Glide XP docking pose of **DTT-S6** in complex with COX-1. (B) Protein-ligand interaction fractions for key residues throughout the 50 ns trajectory. (C) 2D ligand-protein interaction diagram summarizing major contacts (≥ 15% occupancy) between **DTT-S6** and COX-1 observed during the simulation. (D) Timeline of residue-specific interactions with **DTT-S6** over the 50 ns MD simulation, illustrating the persistence and temporal fluctuations of individual contacts.

**Figure S7.** 50 ns MD simulation results for **SC-560** in the COX-1 active site. (A) Glide XP docking pose of SC-560 in complex with COX-1. (B) Protein-ligand interaction fractions for key residues throughout the 50 ns trajectory. (C) 2D ligand-protein interaction diagram summarizing major contacts (≥ 15% occupancy) between SC-560 and COX-1 observed during the simulation. (D) Timeline of residue-specific interactions with SC-560 over the 50 ns MD simulation, illustrating the persistence and temporal fluctuations of individual contacts.

**Figure S8.** 50 ns MD simulation results for **DTT-S6** in the 5-LOX active site. (A) Glide XP docking pose of **DTT-S6** in complex with 5-LOX. (B) Protein-ligand interaction fractions for key residues throughout the 50 ns trajectory. (C) 2D ligand-protein interaction diagram summarizing major contacts (≥ 15% occupancy) between **DTT-S6** and 5-LOX observed during the simulation. (D) Timeline of residue-specific interactions with **DTT-S6** over the 50 ns MD simulation, illustrating the persistence and temporal fluctuations of individual contacts.

**Figure S9.** 50 ns MD simulation results for **DTT-S6** in the 5-LOX allosteric site. (A) Glide XP docking pose of **DTT-S6** in complex with 5-LOX. (B) Protein-ligand interaction fractions for key residues throughout the 50 ns trajectory. (C) 2D ligand-protein interaction diagram summarizing major contacts (≥ 15% occupancy) between **DTT-S6** and 5-LOX observed during the simulation. (D) Timeline of residue-specific interactions with **DTT-S6** over the 50 ns MD simulation, illustrating the persistence and temporal fluctuations of individual contacts.

**Figure S10.** 50 ns MD simulation results for **DTT-S17** in the COX-2 active site. (A) Glide XP docking pose of **DTT-S17** in complex with COX-2. (B) Protein-ligand interaction fractions for key residues throughout the 50 ns trajectory. (C) 2D ligand-protein interaction diagram summarizing major contacts (≥ 15% occupancy) between **DTT-S17** and COX-2 observed during the simulation. (D) Timeline of residue-specific interactions with **DTT-S17** over the 50 ns MD simulation, illustrating the persistence and temporal fluctuations of individual contacts.

**Figure S11.** 50 ns MD simulation results for **DTT-S17** in the 5-LOX active site. (A) Glide XP docking pose of **DTT-S17** in complex with 5-LOX. (B) Protein-ligand interaction fractions for key residues throughout the 50 ns trajectory. (C) 2D ligand-protein interaction diagram summarizing major contacts (≥ 15% occupancy) between **DTT-S17** and 5-LOX observed during the simulation. (D) Timeline of residue-specific interactions with **DTT-S17** over the 50 ns MD simulation, illustrating the persistence and temporal fluctuations of individual contacts.

**Figure S12.** 50 ns MD simulation results for **DTT-S17** in the 5-LOX allosteric site. (A) Glide XP docking pose of **DTT-S17** in complex with 5-LOX. (B) Protein-ligand interaction fractions for key residues throughout the 50 ns trajectory. (C) 2D ligand-protein interaction diagram summarizing major contacts (≥ 15% occupancy) between **DTT-S17** and 5-LOX observed during the simulation. (D) Timeline of residue-specific interactions with **DTT-S17** over the 50 ns MD simulation, illustrating the persistence and temporal fluctuations of individual contacts.

**Table S1.** MetaDrug^TM^-predicted *in silico* toxicity endpoints for the two most promising anticancer derivatives (**DTT-S6** and **DTT-S8**) identified *in vitro*.

| **Toxicity model** | **DTT-S6** | **DTT-S8** |
| --- | --- | --- |
| **AMES^a^ (TP^b^)** | 0.87 (40.33) | 0.21 (32.42) |
| **Cytotoxicity model, pGI_50_^c^ (TP)** | 4.57 (43.16) | 3.96 (36.05) |
| **MRTD^d^ (TP)** | 0.58 (40.81) | 0.45 (40.68) |
| **Anemia^e^ (TP)** | 0.17 (42.41) | 0.11 (35.47) |
| **Carcinogenicity^f^ (TP)** | 0.45 (39.73) | 0.23 (41.31) |
| **Carcinogenicity mouse female^g^ (TP)** | 0.46 (36.03) | 0.39 (41.31) |
| **Carcinogenicity mouse male^h^ (TP)** | 0.50 (36.03) | 0.27 (37.74) |
| **Carcinogenicity rat female^i^ (TP)** | 0.28 (35.02) | 0.27 (41.31) |
| **Carcinogenicity rat male^j^ (TP)** | 0.61 (34.52) | 0.43 (41.31) |
| **Cardiotoxicity^k^ (TP)** | 0.57 (32.29) | 0.18 (31.99) |
| **Genotoxicity^l^ (TP)** | 0.35 (39.27) | 0.33 (36.05) |
| **Hepatotoxicity^m^ (TP)** | 0.55 (39.27) | 0.35 (38.00) |
| **Nephrotoxicity^n^ (TP)** | 0.42 (38.27) | 0.25 (44.36) |
| **Neurotoxicity^o^ (TP)** | 0.70 (36.03) | 0.48 (34.31) |
| **Liver cholestasis^p^ (TP)** | 0.52 (42.41) | 0.50 (35.47) |
| **Liver lipid accumulation^r^ (TP)** | 0.43 (37.50) | 0.22 (34.11) |
| **Liver necrosis^s^ (TP)** | 0.96 (35.90) | 0.18 (36.12) |
| **Liver weight gain^t^ (TP)** | 0.99 (39.27) | 0.36 (37.91) |
| **Kidney necrosis^u^ (TP)** | 0.50 (37.04) | 0.21 (33.33) |
| **Kidney weight gain^v^ (TP)** | 0.40 (42.41) | 0.21 (36.12) |
| **Nephron injury^y^ (TP)** | 0.44 (42.41) | 0.21 (41.31) |
| **SkinSens, EC3^z^ (TP)** | 14.91 (31.72) | 17.49 (29.54) |
| **Nasal pathology^aa^ (TP)** | 0.14 (33.64) | 0.23 (36.81) |
| **Testicular toxicity^ab^ (TP)** | 0.45 (38.56) | 0.33 (40.85) |
| **Pulmonary toxicity^ac^ (TP)** | 0.55 (31.82) | 0.19 (38.01) |
| **Epididymis toxicity^ad^ (TP)** | 0.72 (38.27) | 0.51 (32.03) |

^a^*In silico* Ames mutagenicity prediction model based on the reversion of mutations in the histidine operon of *Salmonella enterica* sv. Typhimurium. The model yields a continuous score between 0 and 1; values ≥ 0.5 are classified as AMES-positive (mutagenic), and values < 0.5 as AMES-negative (non-mutagenic), with lower scores indicating a more favorable profile. Regression model statistics: N = 1780, R^2^ = 0.69, RMSE = 0.29.

^b^Tanimoto Prioritization (TP): The maximum Tanimoto similarity between the query compound (including its predicted metabolites) and any molecule within the QSAR training set. Higher scores reflect greater structural resemblance to the training set and therefore indicate increased reliability of the model predictions

^c^Regression-based cytotoxicity model for the human MCF-7 breast adenocarcinoma cell line, expressed as pGI_50_. A cutoff of pGI_50_ = 6 is applied: values between 6 and 8 denote cytotoxic (toxic) compounds, values < 6 are considered more favorable (less toxic), and values < 3 indicate very low cytotoxicity. Model statistics: N = 1474, R^2^ = 0.90, RMSE = 0.05.

^d^Regression model predicting human MRTD as log(mg/kg body mass/day). The predicted values range from -5 to 3, with a cutoff of 0.5 for interpretation; higher log MRTD values correspond to mildly toxic compounds (higher tolerated doses), whereas lower log MRTD values indicate highly toxic compounds. Model statistics: N = 1209, R^2^ = 0.86, RMSE = 0.42.

^e^Classification model predicting human anemia liability based on *in vivo* data for chemicals and drugs. A predicted probability ≥ 0.5 indicates a positive anemia liability (toxic), whereas values < 0.5 indicate low risk. Model performance: training set N = 324, test set N = 51, sensitivity = 0.82, specificity = 0.90, accuracy = 0.86, MCC = 0.72.

^f^Global rodent carcinogenicity model derived from *in vivo* mouse and rat data (ISSCAN). Predicted probabilities ≥ 0.5 are classified as carcinogenic, whereas values < 0.5 indicate non-carcinogenic compounds. Model performance: training set N = 1210, test set N = 185, sensitivity = 0.96, specificity = 0.90, accuracy = 0.93, MCC = 0.86.

^g^Carcinogenicity prediction model for female mice based on *in vivo* data. Scores ≥ 0.5 are classified as carcinogenic, whereas scores < 0.5 are classified as non-carcinogenic. Model performance: training set N = 640, test set N = 94, sensitivity = 0.90, specificity = 0.93, accuracy = 0.92, MCC = 0.83.

^h^Carcinogenicity prediction model for male mice based on *in vivo* data. Predicted probabilities ≥ 0.5 are classified as positive (carcinogenic), whereas values < 0.5 are classified as negative (non-carcinogenic). Model performance: training set N = 584, test set N = 93, sensitivity = 0.91, specificity = 0.88, accuracy = 0.89, MCC = 0.78.

^i^Carcinogenicity prediction model for female rats. Scores ≥ 0.5 are classified as carcinogenic, whereas scores < 0.5 indicate non-carcinogenic compounds. Model performance: training set N = 667, test set N = 120, sensitivity = 0.90, specificity = 0.96, accuracy = 0.93, MCC = 0.86.,

^j^Carcinogenicity prediction model for male rats. A cutoff value of 0.5 is applied: scores ≥ 0.5 are classified as carcinogenic, whereas scores < 0.5 are classified as non-carcinogenic. Model performance: training set N = 715, test set N = 117, sensitivity = 0.92, specificity = 0.88, accuracy = 0.90, MCC = 0.79.

^k^Cardiotoxicity prediction model based on *in vivo* data from mice, rats, and humans. Predicted probabilities ≥ 0.5 are classified as cardiotoxic, whereas values < 0.5 indicate low cardiotoxicity risk. Model performance: training set N = 143, test set N = 30, sensitivity = 0.80, specificity = 1.00, accuracy = 0.90, MCC = 0.82.

^l^Rodent genotoxicity prediction model based on National Toxicology Program (NTP) data. Scores ≥ 0.5 are classified as genotoxic, whereas scores < 0.5 indicate non-genotoxic compounds. Model performance: training set N = 372, test set N = 86, sensitivity = 0.75, specificity = 0.84, accuracy = 0.79, MCC = 0.59.

^m^Hepatotoxicity prediction model based on *in vivo* data from mice, rats, and humans. A cutoff of 0.5 is applied: predicted probabilities ≥ 0.5 indicate hepatotoxic compounds, whereas values < 0.5 indicate low hepatotoxic risk. Model performance: training set N = 1380, test set N = 231, sensitivity = 0.73, specificity = 0.88, accuracy = 0.81, MCC = 0.62.

^n^General nephrotoxicity prediction model derived from *in vivo* data in mice, rats, and humans. Scores ≥ 0.5 indicate nephrotoxic potential, whereas scores < 0.5 indicate low nephrotoxic risk. Model performance: training set N = 847, test set N = 154, sensitivity = 0.90, specificity = 0.84, accuracy = 0.87, MCC = 0.74.

^o^Neurotoxicity prediction model based on *in vivo* data from mice, rats, and humans. Predicted probabilities ≥ 0.5 are classified as neurotoxic, whereas values < 0.5 are classified as non-neurotoxic. Model performance: training set N = 175, test set N = 34, sensitivity = 0.94, specificity = 0.94, accuracy = 0.94, MCC = 0.88.

^p^Liver cholestasis prediction model based on *in vivo* data from mice, rats, and humans. Scores ≥ 0.5 indicate positive cholestasis liability, whereas scores < 0.5 indicate low risk. Model performance: training set N = 218, test set N = 35, sensitivity = 0.79, specificity = 0.67, accuracy = 0.74, MCC = 0.46.

^r^Prediction model for hepatic lipid accumulation in mice, rats, and humans. Probabilities ≥ 0.5 are classified as positive for liver lipid accumulation, whereas values < 0.5 are classified as negative. Model performance: training set N = 172, test set N = 28, sensitivity = 0.80, specificity = 0.85, accuracy = 0.82, MCC = 0.64.

^s^Prediction model for hepatic necrosis in mice, rats, and humans. A cutoff of 0.5 is applied: scores ≥ 0.5 are classified as positive for liver necrosis, whereas scores < 0.5 are classified as negative. Model performance: training set N = 300, test set N = 57, sensitivity = 0.91, specificity = 0.91, accuracy = 0.91, MCC = 0.82.

^t^Prediction model for liver weight gain in rodent studies. Probabilities ≥ 0.5 indicate compounds associated with increased liver weight, whereas values < 0.5 indicate absence of this effect. Model performance: training set N = 292, test set N = 52, sensitivity = 1.00, specificity = 1.00, accuracy = 1.00, MCC = 1.00.

^u^Renal necrosis prediction model based on *in vivo* data from mice, rats, and humans. Predicted probabilities ≥ 0.5 are classified as positive for renal necrosis, whereas values < 0.5 are classified as negative. Model performance: training set N = 221, test set N = 42, sensitivity = 0.96, specificity = 1.00, accuracy = 0.98, MCC = 0.95.

^v^Prediction model for kidney weight gain in rodent studies. Predicted probabilities ≥ 0.5 identify compounds associated with increased kidney weight, whereas values < 0.5 indicate absence of this liability. Model performance: training set N = 240, test set N = 49, sensitivity = 0.95, specificity = 1.00, accuracy = 0.98, MCC = 0.96.

^y^Nephron injury prediction model based on in vivo data from mice, rats, and humans. Predicted probabilities ≥ 0.5 are classified as positive for nephron injury, whereas values < 0.5 are classified as negative. Model performance: training set N = 598, test set N = 109, sensitivity = 0.91, specificity = 1.00, accuracy = 0.96, MCC = 0.93.

^z^Regression model for skin sensitization based on the effective concentration EC3 (%). Values > 10 correspond to weak-to-moderate skin sensitizers, whereas lower EC3 values indicate stronger sensitizers with higher sensitization potency. Model statistics: N = 89, R^2^ = 0.67, RMSE = 22.56.

^aa^Nasal pathology prediction model based on *in vivo* data from mice, rats, and humans. Predicted probabilities ≥ 0.5 are classified as positive for nasal toxicity/pathology, whereas values < 0.5 indicate low risk. Model performance: training set N = 246, test set N = 47, sensitivity = 1.00, specificity = 0.93, accuracy = 0.96, MCC = 0.92.

^ab^Testicular toxicity prediction model derived from *in vivo* data in mice, rats, and humans. Predicted probabilities ≥ 0.5 are classified as positive for testicular toxicity, whereas values < 0.5 indicate low risk. Model performance: training set N = 439, test set N = 88, sensitivity = 0.81, specificity = 0.85, accuracy = 0.83, MCC = 0.66.

^ac^Pulmonary toxicity prediction model derived from *in vivo* data in mice, rats, and humans. Scores ≥ 0.5 are interpreted as positive for pulmonary toxicity liability, whereas scores < 0.5 indicate low risk. Model performance: training set N = 482, test set N = 87, sensitivity = 0.89, specificity = 0.88, accuracy = 0.89, MCC = 0.77.

^ad^Epididymal toxicity prediction model based on *in vivo* data from mice, rats, and humans. Predicted probabilities ≥ 0.5 are classified as positive for epididymal toxicity, whereas values < 0.5 are classified as negative. Model performance: training set N = 252, test set N = 42, sensitivity = 0.90, specificity = 0.86, accuracy = 0.88, MCC = 0.76.

**References**

Ahmadi, M., Bekeschus, S., Weltmann, K.-D., von Woedtke, T., & Wende, K. (2022). Non-steroidal anti-inflammatory drugs: Recent advances in the use of synthetic COX-2 inhibitors. *RSC Medicinal Chemistry, 13*(5), 471-496. <https://doi.org/10.1039/d1md00280e>

Colovos, C., & Yeates, T. O. (1993). Verification of protein structures: Patterns of nonbonded atomic interactions. *Protein Science, 2*(9), 1511-1519. <https://doi.org/10.1002/pro.5560020916>

Faki, Y., & Er, A. (2021). Different chemical structures and physiological/pathological roles of cyclooxygenases. *Rambam Maimonides Medical Journal*, *12*(1), e0003. <https://doi.org/10.5041/RMMJ.10426>

Gilbert, N. C., Gerstmeier, J., Schexnaydre, E. E., Börner, F., Garscha, U., Neau, D. B., Werz, O., & Newcomer, M. E. (2020). Structural and mechanistic insights into 5-lipoxygenase inhibition by natural products. *Nature Chemical Biology, 16*(7), 783-790. <https://doi.org/10.1038/s41589-020-0544-7>

Guillon, C., Robert, X., & Gouet, P. (2024). “It’s only a model”: When protein structure predictions need experimental validation, the case of the HTLV-1 Tax protein. *Pathogens, 13*(3), 241. <https://doi.org/10.3390/pathogens13030241>

Limongelli, V., Bonomi, M., Marinelli, L., Gervasio, F. L., Cavalli, A., Novellino, E., & Parrinello, M. (2010). Molecular basis of cyclooxygenase enzymes (COXs) selective inhibition. *Proceedings of the National Academy of Sciences of the United States of America, 107*(12), 5411-5416. <https://doi.org/10.1073/pnas.0913377107>

Liu, Y., Wang, K., Cao, F., Gao, N., & Li, W. (2024). Interactions between inhibitors and 5-lipoxygenase: Insights from Gaussian accelerated molecular dynamics and Markov state models. *International Journal of Molecular Sciences, 25*(15), 8295. <https://doi.org/10.3390/ijms25158295>

Md Idris, M. H., Mohd Amin, S. N., Mohd Amin, S. N., Nyokat, N., Khong, H. Y., Selvaraj, M., Zakaria, Z. A., Shaameri, Z., Hamzah, A. S., Teh, L. K., & Salleh, M. Z. (2022). Flavonoids as dual inhibitors of cyclooxygenase-2 (COX-2) and 5-lipoxygenase (5-LOX): Molecular docking and *in vitro* studies. *Beni-Suef University Journal of Basic and Applied Sciences, 11*, Article 117. <https://doi.org/10.1186/s43088-022-00296-y>

Nguyen, H. T., Vu, T. Y., Vijay Kumar, A., Hoang, V. N. H., My, P. T. N., Mandal, P. S., & Tatipamula, V. B. (2021). *N*-Aryl iminochromenes inhibit cyclooxygenase enzymes via π-π stacking interactions and present a novel class of anti-inflammatory drugs. *RSC Advances*, *11*(47), 29385-29393. <https://doi.org/10.1039/d1ra04407a>

Orlando, B. J., & Malkowski, M. G. (2016). Crystal structure of rofecoxib bound to human cyclooxygenase-2. *Acta Crystallographica Section F: Structural Biology Communications, 72*(10), 772-776. <https://doi.org/10.1107/S2053230X16014230>

Rakonjac Ryge, M., Tanabe, M., Provost, P., Persson, B., Chen, X., Funk, C. D., Rinaldo-Matthis, A., Hofmann, B., Steinhilber, D., Watanabe, T., Samuelsson, B., & Rådmark, O. (2014). A mutation interfering with 5-lipoxygenase domain interaction leads to increased enzyme activity. *Archives of Biochemistry and Biophysics, 545*, 179-185. <https://doi.org/10.1016/j.abb.2014.01.017>

Rouzer, C. A., & Marnett, L. J. (2020). Structural and chemical biology of the interaction of cyclooxygenase with substrates and non-steroidal anti-inflammatory drugs. *Chemical Reviews*, *120*(15), 7592-7641. <https://doi.org/10.1021/acs.chemrev.0c00215>

Shin, W. H., Kang, X., Zhang, J., & Kihara, D. (2017). Prediction of local quality of protein structure models considering spatial neighbors in graphical models. *Scientific Reports, 7*, 40629. <https://doi.org/10.1038/srep40629>

Smith, C. J., Zhang, Y., Koboldt, C. M., Muhammad, J., Zweifel, B. S., Shaffer, A., Talley, J. J., Masferrer, J. L., Seibert, K., & Isakson, P. C. (1998). Pharmacological analysis of cyclooxygenase-1 in inflammation. *Proceedings of the National Academy of Sciences of the United States of America, 95*(22), 13313-13318. <https://doi.org/10.1073/pnas.95.22.13313>

Tenorio, J. C. B., Heikal, M. F., Kafle, A., Saichua, P., & Suttiprapa, S. (2024). Benzimidazole resistance-associated mutations improve the in silico dimerization of hookworm tubulin: An additional resistance mechanism. *Veterinary World, 17*(12), 2736-2746. <https://doi.org/10.14202/vetworld.2024.2736-2746>

Waterhouse, A., Bertoni, M., Bienert, S., Studer, G., Tauriello, G., Gumienny, R., Heer, F. T., de Beer, T. A. P., Rempfer, C., Bordoli, L., Lepore, R., & Schwede, T. (2018). SWISS-MODEL: Homology modelling of protein structures and complexes. *Nucleic Acids Research, 46*(W1), W296-W303. <https://doi.org/10.1093/nar/gky427>

Wlodawer, A. (2017). Stereochemistry and validation of macromolecular structures. In *Methods in Molecular Biology* (Vol. 1607, pp. 595-610). Humana Press. <https://doi.org/10.1007/978-1-4939-7000-1_24>

Wu, N., Zhang, R., Peng, X., Fang, L., Chen, K., & Jestilä, J. S. (2024). Elucidation of protein–ligand interactions by multiple trajectory analysis methods. *Physical Chemistry Chemical Physics, 26*(8), 6903-6915. <https://doi.org/10.1039/d3cp03492e>

**Figure S13.** FT-IR Spectra of **ADT-OMe**

**Figure S14.** ^1^H NMR spectra of **ADT-OMe**

**Figure S15.** FT-IR Spectra of **ADT-OH**

**Figure S16.** ^1^H NMR spectra of **ADT-OH**

**Figure S17.** ^13^C NMR spectra of **ADT-OH**

**Figure S18.** FT-IR Spectra of **DTT-S1**

**Figure S19.** ^1^H NMR spectra of **DTT-S1**

 **Figure S20.** ^13^C NMR spectra of **DTT-S1**

**Figure S21.** MS spectra of **DTT-S1**

**Figure S22.** FT-IR Spectra of **DTT-S2**

**Figure S23.** ^1^H NMR spectra of **DTT-S2**

 **Figure S24.** ^13^C NMR spectra of **DTT-S2**

**Figure S25.** MS spectra of **DTT-S2**

**Figure S26.** FT-IR Spectra of **DTT-S3**

**Figure S27.** ^1^H NMR spectra of **DTT-S3**

**Figure S28**. ^13^C NMR spectra of **DTT-S3**

**Figure S29.** MS spectra of **DTT-S3**

**Figure S30.** FT-IR Spectra of **DTT-S4**

**Figure S31.** ^1^H NMR spectra of **DTT-S4**

**Figure S32.** ^13^C NMR spectra of **DTT-S4**

**Figure S33.** MS spectra of **DTT-S4**

**Figure S34.** FT-IR Spectra of **DTT-S5**

**Figure S35.** ^1^H NMR spectra of **DTT-S5**

**Figure S36.** ^13^C NMR spectra of **DTT-S5**

**Figure S37.** MS spectra of **DTT-S5**

**Figure S38.** FT-IR Spectra of **DTT-S6**

**Figure S39.** ^1^H NMR spectra of **DTT-S6**

**Figure S40.** ^13^C NMR spectra of **DTT-S6**

**Figure S41.** MS spectra of **DTT-S6**

**Figure S42.** FT-IR Spectra of **DTT-S7**

**Figure S43.** ^1^H NMR spectra of **DTT-S7**

**Figure S44.** ^13^C NMR spectra of **DTT-S7**

 **Figure S45.** MS spectra of **DTT-S7**

**Figure S46.** FT-IR Spectra of **DTT-S8**

**Figure S47.** ^1^H NMR spectra of **DTT-S8**

**Figure S48.** ^13^C NMR spectra of **DTT-S8**

**Figure S49.** MS spectra of **DTT-S8**

 **Figure S50.** FT-IR Spectra of **DTT-S9**

**Figure S51.** ^1^H NMR spectra of **DTT-S9**

**Figure S52.** ^13^C NMR spectra of **DTT-S9**

**Figure S53.** MS spectra of **DTT-S9**

**Figure S54.** FT-IR Spectra of **DTT-S10**

**Figure S55.** ^1^H NMR spectra of **DTT-S10**

**Figure S56.** ^13^C NMR spectra of **DTT-S10**

**Figure S57.** MS spectra of **DTT-S10**

**Figure S58.** FT-IR Spectra of **DTT-S11**

**Figure S59.** ^1^H NMR spectra of **DTT-S11**

**Figure S60.** ^13^C NMR spectra of **DTT-S11**

**Figure S61.** MS spectra of **DTT-S11**

**Figure S62.** FT-IR Spectra of **DTT-S12**

**Figure S63.** ^1^H NMR spectra of **DTT-S12**

**Figure S64.** ^13^C NMR spectra of **DTT-S12**

**Figure S65.** MS spectra of **DTT-S12**

**Figure S66.** FT-IR Spectra of **DTT-S13**

**Figure S67.** ^1^H NMR spectra of **DTT-S13**

**Figure S68.** ^13^C NMR spectra of **DTT-S13**

**Figure S69.** MS spectra of **DTT-S13**

**Figure S70.** FT-IR Spectra of **DTT-S14**

**Figure S71.** ^1^H NMR spectra of **DTT-S14**

 **Figure S72.** ^13^C NMR spectra of **DTT-S14**

**Figure S73.** MS spectra of **DTT-S14**

**Figure S74.** FT-IR Spectra of **DTT-S15**

**Figure S75.** ^1^H NMR spectra of **DTT-S15**

**Figure S76.** ^13^C NMR spectra of **DTT-S15**

**Figure S77.** MS spectra of **DTT-S15**

**Figure S78.** FT-IR Spectra of **DTT-S16**

**Figure S79.** ^1^H NMR spectra of **DTT-S16**

**Figure S80.** ^13^C NMR spectra of **DTT-S16**

**Figure S81.** MS spectra of **DTT-S16**

**Figure S82.** FT-IR Spectra of **DTT-S17**

**Figure S83.** ^1^H NMR spectra of **DTT-S17**

**Figure S84.** ^13^C NMR spectra of **DTT-S17**

**Figure S85.** MS spectra of **DTT-S17**

**Figure S86.** FT-IR Spectra of **DTT-S18**

**Figure S87.** ^1^H NMR spectra of **DTT-S18**

**Figure S88.** ^13^C NMR spectra of **DTT-S18**

**Figure S89.** MS spectra of **DTT-S18**

**Table S2.** The purity of compounds **DTT-S1-18**

| **Compound** | **Retention Time (min)** | **Purity (%)** |
| --- | --- | --- |
| **DTT-S1** | 8.61 | 100 |
| **DTT-S2** | 9.93 | 96.46 |
| **DTT-S3** | 8.12 | 100 |
| **DTT-S4** | 6.36 | 95.34 |
| **DTT-S5** | 5.57 | 98.49 |
| **DTT-S6** | 7.34 | 97.28 |
| **DTT-S7** | 5.66 | 96.04 |
| **DTT-S8** | 5.56 | 100 |
| **DTT-S9** | 4.72 | 98.20 |
| **DTT-S10** | 5.89 | 97.42 |
| **DTT-S11** | 5.92 | 97.52 |
| **DTT-S12** | 5.92 | 97.90 |
| **DTT-S13** | 5.02 | 100 |
| **DTT-S14** | 4.20 | 95.33 |
| **DTT-S15** | 6.16 | 95.80 |
| **DTT-S16** | 5.01 | 95.54 |
| **DTT-S17** | 5.64 | 100 |
| **DTT-S18** | 9.45 | 99.40 |

**Figure S90.** HPLC chromatogram of **DTT-S1**

**Figure S91.** HPLC chromatogram of **DTT-S2**

**Figure S92.** HPLC chromatogram of **DTT-S3**

**Figure S93.** HPLC chromatogram of **DTT-S4**

**Figure S94.** HPLC chromatogram of **DTT-S5**

**Figure S95.** HPLC chromatogram of **DTT-S6**

**Figure S96.** HPLC chromatogram of **DTT-S7**

**Figure S97**. HPLC chromatogram of **DTT-S8**

**Figure S98.** HPLC chromatogram of **DTT-S9**

**Figure S99.** HPLC chromatogram of **DTT-S10**

**Figure S100.** HPLC chromatogram of **DTT-S11**

**Figure S101.** HPLC chromatogram of **DTT-S12**

**Figure S102.** HPLC chromatogram of **DTT-S13**

**Figure S103**. HPLC chromatogram of **DTT-S14**

**Figure S104.** HPLC chromatogram of **DTT-S15**

**Figure S105.** HPLC chromatogram of **DTT-S16**

**Figure S106.** HPLC chromatogram of **DTT-S17**

**Figure S107.** HPLC chromatogram of **DTT-S18**

**Figure S108.** IC_50_ values (μM) of compounds (A) **DTT-S6** and (B) **DTT-S8** on MCF-7 cell line

**Figure S109.** IC_50_ values (μM) of compounds (A) **DTT-S6** and (B) **DTT-S8** on HUVEC cell line
